# Inconsistency in UK Biobank Event Definitions From Different Data Sources and Its Impact on Bias and Generalizability: A Case Study of Venous Thromboembolism

**DOI:** 10.1093/aje/kwad232

**Published:** 2023-11-17

**Authors:** Emily Bassett, James Broadbent, Dipender Gill, Stephen Burgess, Amy M Mason

**Keywords:** bias, deep vein thrombosis, event definition, generalizability, pulmonary embolism, representativeness, sociodemographic characteristics, UK Biobank, venous thromboembolism

## Abstract

The UK Biobank study contains several sources of diagnostic data, including hospital inpatient data and data on self-reported conditions for approximately 500,000 participants and primary-care data for approximately 177,000 participants (35%). Epidemiologic investigations require a primary disease definition, but whether to combine data sources to maximize statistical power or focus on only 1 source to ensure a consistent outcome is not clear. The consistency of disease definitions was investigated for venous thromboembolism (VTE) by evaluating overlap when defining cases from 3 sources: hospital inpatient data, primary-care reports, and self-reported questionnaires. VTE cases showed little overlap between data sources, with only 6% of reported events for persons with primary-care data being identified by all 3 sources (hospital, primary-care, and self-reports), while 71% appeared in only 1 source. Deep vein thrombosis–only events represented 68% of self-reported VTE cases and 36% of hospital-reported VTE cases, while pulmonary embolism–only events represented 20% of self-reported VTE cases and 50% of hospital-reported VTE cases. Additionally, different distributions of sociodemographic characteristics were observed; for example, patients in 46% of hospital-reported VTE cases were female, compared with 58% of self-reported VTE cases. These results illustrate how seemingly neutral decisions taken to improve data quality can affect the representativeness of a data set.

## Abbreviations

DVT: deep vein thrombosis
PE: pulmonary embolism
VTE: venous thromboembolism

Venous thromboembolism (VTE) is a condition that occurs when a blood clot forms inside the veins, preventing blood flow. Its incidence is roughly 100 events per 100,000 person-years (1, 2). Approximately two-thirds of cases are deep vein thrombosis (DVT) (3–5), where the blood clot forms in a deep vein, typically the pelvis, thigh, or lower leg. A third of cases are pulmonary embolism (PE), which occurs when the clot breaks loose and travels to the lungs (3–5). In rare cases, thrombosis may occur in other veins.

Factors associated with greater risk of VTE include obesity (6), increased height (7), positive smoking status (8), hypertension (9), increased social deprivation (10, 11), more years of education (9), immobilization (12), surgery (13), use of hormone replacement therapy or oral contraceptives (14, 15), and pregnancy (16). Risk of VTE also increases with age, as does the proportion of VTEs that are PEs (17). There is little consistent evidence for overall differences in VTE risk by sex: There are reports of higher rates for men (2, 9, 17), no significant difference (18, 19), and higher rates for women (3, 20–22) when combining across all ages. However, there may be different patterns of risk across the lifetime, with risk being higher during the reproductive years among women and higher in old age among men (2, 3, 20), and men being at higher risk of recurrent events (23, 24). VTE risk is higher for individuals of African ancestry than for those of European ancestry (25) and higher for individuals of European ancestry than for those of Hispanic and Asian ancestry (26, 27).

Previous studies on VTE in the UK Biobank have used a combination of self-reported physician diagnosis of DVT or PE, details from hospital inpatient records, and death certificates, or a subset of these sources (28–30). Details from primary-care records in the UK Biobank are used less often, as they are not available for the entire cohort. Other studies have used different sources to determine VTE cases. A 23andMe study used self-reported VTEs alone (31), while a large Norwegian study used a combination of inpatient and outpatient hospital records (3). Because studies do not typically break down results by source of diagnosis report, it is unclear how much different reporting sources could affect the number and sociodemographic makeup of identified cases.

Electronic health records used for research are susceptible to “informed presence bias.” Patients do not appear in health records at random, but are influenced by the symptoms they have and how well they can communicate them to clinicians (32, 33). Clinician suspicion determines who is scored for suspected DVT/PE. A probability assessment using the modified DVT and PE Wells scores determines who then gets to access further tests such as D-dimer measurement, ultrasonography, and radiological imaging (34). Currently in the United Kingdom’s National Health Service, individuals with DVT and low-risk PE can be treated as outpatients (35), while those with high-risk PE would be admitted to a hospital as inpatients—this could influence which data sources record a VTE and whose VTEs get recorded.

Our aim in this investigation is to determine how using different sources of data may affect VTE case populations within the UK Biobank. We will do this by considering how closely reports of VTE from different data sources correspond and whether the populations reported as cases are similar. We will not consider any specific reporting method as a “gold standard” of truth to determine the accuracy of other methods, nor will we attempt to estimate VTE incidence in the general UK population. Instead, we will compare how similar each definition is to the others within the UK Biobank.

## METHODS

### Study participants

The UK Biobank is a large prospective cohort study containing diagnostic data for 503,317 participants aged 37–73 years who were recruited across England, Scotland, and Wales between 2006 and 2010.

### Data sources within the UK Biobank

#### Self-reported outcomes.

At enrollment and resurvey, participants answered a touch-screen questionnaire, including specific questions about prior physician diagnoses of blood clots in the leg or lungs as well as more general questions about serious medical conditions. These were followed up with a verbal interview (36). Where participants were not certain about prior diagnoses, their responses were matched where possible to health conditions in a coding tree by a medical professional (37). Self-reported VTEs were coded as either DVT, PE, or other VTE.

#### Hospital data.

*International Classification of Diseases, Ninth Revision*, and *International Classification of Diseases, Tenth Revision*, coded hospital inpatient episodes were obtained from the Hospital Episode Statistics provider for England, the Patient Episode Data for Wales, and the Scottish Morbidity Records for Scotland (38). These data sets contain information on admission and discharge, operations, diagnoses, maternity care, and psychiatric care. Main and secondary diagnoses throughout the patient’s admission are recorded. These data are only available within the UK Biobank for patients who are admitted to the hospital and occupy a bed.

#### Death certificate data.

*International Classification of Diseases, Ninth Revision*, coded national death registry data were obtained from the Health and Social Care Information Centre (now NHS England) for England and Wales and the Information Services Department for Scotland (39). This includes primary and secondary causes of death determined by a physician who attended the patient in their last illness or a coroner (40).

#### Primary-care records.

Primary-care data were captured for 230,000 participants, covering records from selected general practice services in England, Scotland, and Wales (41). We took a subset of 177,363 participants that ensured continuous coverage overlapping with their recruitment into the UK Biobank. Details on choices made can be found in Web Appendix 1 (available at https://doi.org/10.1093/aje/kwad232).

### Event definitions

VTE cases were determined using the 4 data sources: death certificate data, hospital data, self-reported outcomes, and primary-care records (in the primary-care cohort only). We considered events reported by each data source in turn, as well as a combined outcome including events reported by any data source.

VTE cases were broken down into PE and DVT via matching to any of the codes in Web Table 1. If a participant matched to VTE but not to PE or DVT, they were classed as “other VTE.”

### Medication use

One concern about the self-reported outcomes is that case definitions may be much less accurate. To investigate this concern, we considered whether patterns of relevant medication use were similar between the cases reported via different sources.

VTEs are often treated with anticoagulants. While warfarin is not recommended as the first line treatment in the current UK guidelines, the standard of care prior to 2020 was a low-molecular-weight heparin bridge followed by warfarin (34).

There are 2 sources of general medication usage data within the UK Biobank. One is self-reported data, collecting lists of all regularly taken prescription medications during the touch-screen questionnaire and verbal interview (data field 20003) (36). The other source is the primary-care records prescription data, which are available only for the cohort with primary-care data (data field 42039). Matching on drug names was undertaken to identify participants who had taken either any anticoagulant or warfarin at some point (details of matching are shown in Web Table 2).

### Statistical methods

We cross-tabulated the events in both the full UK Biobank sample and the cohort with available primary-care data. In both groups, we compared anticoagulant medication use and demographic data defined by the various data sources. The variables we compared were age at baseline, sex, smoking status, ethnicity, body mass index (weight (kg)/height (m)^2^), current employment status, highest level of education, history of manual or shift work, Townsend deprivation index (a greater score reflects more deprivation), house ownership, and car ownership. For participants in England, we also looked at the Index of Multiple Deprivation and the scores that determine the Index of Multiple Deprivation.

**Table 1:** Demographic Characteristics of UK Biobank Participants at Recruitment, 2006–2010

**Characteristic**	**All Participants** **(*n* = 502,520)**		**Primary-Care Cohort** **(*n* = 177,358)**	
	**%**	**Mean (SD)**	**%**	**Mean (SD)**
Female sex	54.4		54.5	
Age, years		57.0 (8.1)		57.2 (8.0)
White race/ethnicity	94.6		95.7	
Assessment center				
Wales	4.1		10.3	
Scotland	7.1		12.6	
London	13.7		6.8	
Unemployment	43.1		44.1	
Manual work^a^	7.7		7.9	
Higher education	60.2		59.8	
Townsend deprivation index		−1.29 (3.1)		−1.4 (3.0)
Owning one’s house outright	51.5		52.9	
≥2 cars in household	48.8		48.3	
Shift work^b^	5.6		5.6	
Body mass index^c^				
Women		27.1 (5.2)		27.2 (5.2)
Men		27.8 (4.2)		27.9 (4.3)
Current smoking				
Women	8.9		8.7	
Men	12.5		12.1	

Abbreviation: SD, standard deviation.

^a^ Answered “usually” or “always” to the question, “Does your work involve heavy manual or physical work?”.

^b^ Answered “usually” or “always” to the question, “Does your work involve shift work?”.

^c^ Weight (kg)/height (m)^2^.

Proportional Venn diagrams were plotted to obtain a visual understanding of the various overlaps of cases. Agreement between the methods was evaluated using the Fleiss κ value between all of the sets and Cohen’s κ pairwise between each method. Kappa coefficients less than 0.6 are taken to indicate inadequate agreement, in line with recommendations for health-related studies; those with 0.6 ≤ κ < 0.8 are taken as moderate agreement, with 0.8 ≤ κ < 0.9 as strong agreement, and  0.9 or greater as almost perfect agreement (42).

## RESULTS

### Study population

Table 1 contains a summary of the overall demographic characteristics of the UK Biobank participants. The defined primary-care cohort reproduced the known biases within the UK Biobank data set—there was a “healthy volunteer bias,” with participants being more likely to be older, to be female, to have a lower body mass index, to smoke less, to live in less socioeconomically deprived areas, and to have a greater rate of higher education (education after age 18 years) than the average person in the United Kingdom (43) (Web Table 3). The primary-care cohort had a similar sex and age balance, a slightly greater proportion of White participants, higher rates of unemployment, and lower rates of higher education than the full data set.

### Event definitions in the full UK Biobank sample

No single data source captured all VTE cases, and the percentage captured by different methods varied by case type. Taking a report from any source as a case and breaking cases down by subdiagnosis, 13% of participants had both PE and DVT, 54% had DVT only, 30% had PE only, and 3% had a VTE that fit into neither category.

**Figure 1:**
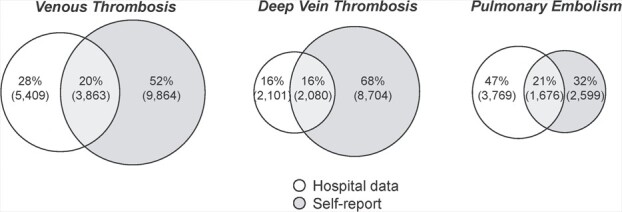
Proportional overlap in venous thromboembolism cases between different data sources in all UK Biobank participants, 2006–2010. In Web Figure 1, these are expanded further into events occurring prior to and after registration in the UK Biobank.

There was little agreement between self-report data and inpatient hospital data (κ = 0.32). Only 20.2% of VTE cases were reported by both sources (Figure 1), while 79.8% appeared only in a single source (51.5% appeared only in self-reports and 28.3% appeared only in hospital data). There was a larger overlap of hospital events being self-reported when we restricted the data to prevalent events, but we did not see a matching hospital report for the majority of incident self-reported events (see Web Figure 1).

The data from death certificates did not add any additional clarity (Web Tables 4 and 5, Web Figures 2 and 3). There were 741 cases of VTE identified from primary and secondary causes of death, of which 388 cases did not appear in another data source. Due to the small proportion of cases identified through this method (approximately 4%), we did not analyze death certificate data further.

Considering the 2 subdiagnoses (DVT and PE), there was a difference in the reporting source by subdiagnosis: More DVTs were only self-reported (67.6%) than were in hospital records only (16.3%) or both in the hospital records and self-reported (16.1%), whereas PEs were most likely to be in hospital records only (46.9%), although nearly a third appeared only as self-reports (32.3%).

**Figure 2:**
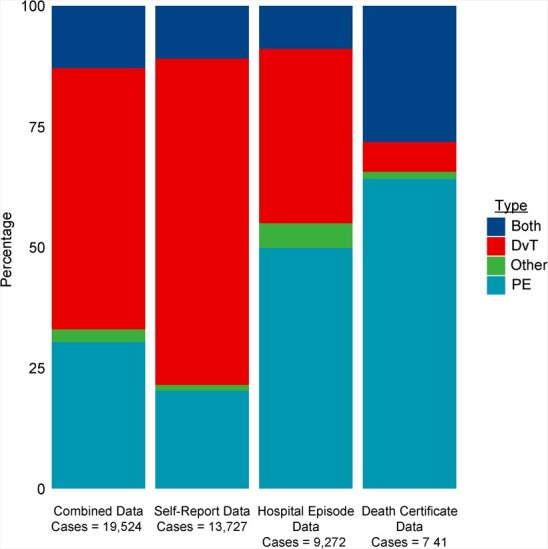
Proportions of deep vein thrombosis (DVT) and pulmonary embolism (PE) cases among identified venous thromboembolism cases when cases were ascertained from different sources, UK Biobank, 2006–2010.

The proportion of DVT to PE events also varied with the data source (Figure 2). If we considered only hospital data, 50% of events were PE only, 36% were DVT only, and 9% were both, whereas when taking self-reported outcomes as the data source, 20% of events were PE only, 68% were DVT only, and 11% were both.

We also saw variation in the demographic characteristics of the identified cases (Table 2). For example, using only hospital data, the case population was 45.7% female, while using the self-reported data the case population was 58.3% female.

**Table 2:** Demographic Comparison Between Case Populations Defined via Different Data Sources, UK Biobank, 2006–2010^a^

	**Data Source**						***P* for Difference Between Self-Reported and Hospital Data**
	**Hospital Data** **(*n* = 9,272)**		**Primary-Care Data** **(*n* = 1,441)**		**Self-Reported** **(*n* = 13,727)**		
**Variable**	**%**	**Mean (SD)**	**%**	**Mean (SD)**	**%**	**Mean (SD)**	
Female sex	45.7		54.0		58.3		<0.0001
Age, years		60.3 (7.2)		60.1 (7.2)		59.7 (7.4)	<0.0001
White race/ethnicity	96.5		97.3		96.0		0.051
Assessment center							
Wales	3.9		14.1		4.9		0.0003
Scotland	7.0		11.7		5.8		0.0002
London	11.4		5.3		11.7		0.486
Not working (retired or unemployed)	60.4		61.1		59.9		0.448
Higher education^b^	52.7		52.4		53		0.655
Current smoker	12.2		12.3		12.4		0.652
Work history							
Heavy manual work^c^	6.5		6.4		5.9		0.063
Shift work^d^	4.6		4.4		4.5		0.721
Townsend deprivation index		−0.89 (3.31)		−1.07 (3.17)		−0.93 (3.27)	0.365
Owning one’s house outright	56.8		57.5		55.0		0.007
>1 car in household	40.7		41.2		42.4		0.010
Body mass index^e^		29.4 (5.6)		29.2 (5.6)		29.2 (5.7)	0.009

Abbreviation: SD, standard deviation.

^a^ A larger version of this table can be seen as Web Table 11. *P* values are reported for independent sample *t* tests for continuous variables and χ^2^ tests of proportions for binary variables.

^b^ Answered >18 years to the question, "At what age did you complete your continuous full time education?" or selected "college or university degree" as a response to the question, "Which of the following qualifications do you have?".

^c^ Answered “usually” or “always” to the question, “Does your work involve heavy manual or physical work?”.

^d^ Answered “usually” or “always” to the question, “Does your work involve shift work?”.

^e^ Weight (kg)/height (m)^2^.

### Event definitions in primary-care cohort

**Figure 3:**
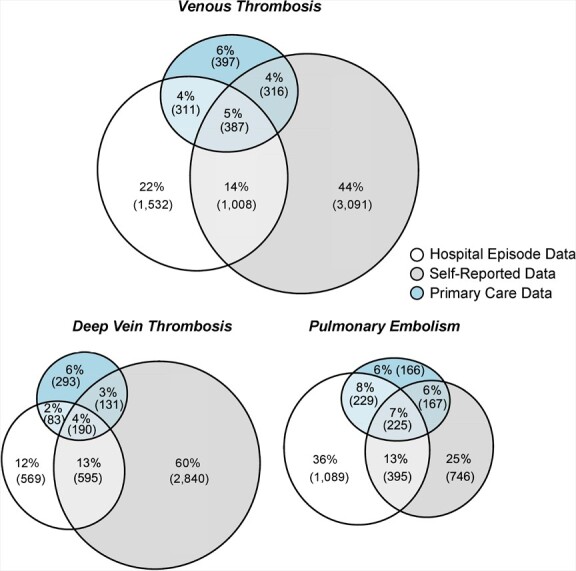
Proportional overlap in venous thromboembolism cases between different data sources in UK Biobank participants with primary-care data, 2006–2010. In Web Figures 4 and 5, these are expanded further into events occurring prior to and after registration in the UK Biobank.

The primary-care cohort within the UK Biobank showed patterns of case overlap similar to those of the full participant group (Figure 3). Adding the additional cases from the primary-care records did not explain many of the undocumented self-reported VTE events and added an additional set of otherwise uncaptured outcomes.

The highest agreement was between hospital data and self-reported data (κ = 0.33), but this was still inadequate in terms of concordance. Primary-care data had slightly more agreement with hospital data than self-reported data (κ = 0.29 vs. 0.21). Only 5.5% of VTE cases were reported by all 3 sources, while 71.3% appeared only in a single source: 43.9% appeared only in self-reports, 21.8% appeared only in hospital data, and 5.6% appeared only in primary-care reports. Splitting the data into prior registration and postregistration, there was a clear time-period effect due to the lack of self-reports postregistration for many participants and the sparsity of hospital reports prior to registration. In all cases, the primary-care data and the hospital data had little overlap (Web Figures 4 and 5, Web Tables 6–8).

There was a difference in the source of the report for the subdiagnoses: Most DVTs were only self-reported (60.4%), while more PEs were in hospital records only (36.1%) than in any other category. There was slightly better agreement between sources for PE (κ = 0.33–0.35 when comparing hospital data, primary-care data, and self-reports) than for DVT (κ = 0.14–0.27). (See Web Table 9.)

### Anticoagulant usage in the primary-care cohort

There were different pattens of reported anticoagulant use between the different case groups, but all had much higher rates than the control group (Web Table 10). Patients whose VTE was identified using only hospital data were more likely to have a record of anticoagulant drug use at some point in their primary-care records (64.7% used some sort of anticoagulant; 50.4% were on warfarin), whereas those identified via primary-care records only had much lower use (37.9% and 26.8%, respectively). Self-reported-only cases fell between these 2 groups (51.2% and 33.2%, respectively). In contrast, anticoagulant drug use among controls (i.e., individuals with no reported VTE event from any source) was much lower (18.9% and 2.5%, respectively). This provided an indication that there were likely to be true VTE events among the self-reported-only cases. Self-reported rates of anticoagulant use were much lower but more consistent between definitions (Web Figure 6).

### Differences in sociodemographic characteristics between cases from each data source

The self-reported cases were younger and more likely to be female than the hospital data cases. They were more likely to have been assessed at the UK Biobank centers in Wales and less likely to have been assessed in Scotland. There were also differences between these 2 case groups in terms of mean body mass index, house ownership, and multiple car ownership. The cases identified by primary-care data were somewhere between the other 2 case groups in terms of both sex and age, with lower levels of deprivation and higher rates of house and multiple car ownership.

## DISCUSSION

Our investigation found that using different data sources in the UK Biobank results in substantial differences in the number, balance, and sociodemographic characteristics of VTE cases considered. None of the data sources had good agreement with each other. The majority of DVT events appeared only as self-reported outcomes. For PE, the largest group of events was reports from hospital data only. One likely reason for this is severity, with DVTs being more likely to be treated in outpatient settings (35) while PE is more often life-threatening, resulting in hospitalization. Hospital reports constituted the majority of postregistration events in the study, while the majority of events prior to registration were self-reported. However, this was not a pure effect of time period, as there were self-reports after registration that were not seen in hospital records and hospital reports before registration than were not-self reported. For both diseases, only a small proportion of participants were detected by multiple data sources as having an event. This suggests a need to be attentive to how use of different data sources may influence case definition and composition.

Large studies of patient characteristics affect our perception of diseases: For example, studies claiming that VTE predominantly affects male or female patients probably affect physicians’ perceptions of reported symptoms, as has been seen for cardiovascular disease (44) and depression (45). This can affect how readily they diagnose future patients. As a result, decisions drawn from biased data can lead to greater health inequality, as has previously been observed for algorithmic decisions (46, 47). Future studies can also be biased by these perceptions, with well-meaning and seemingly neutral decisions taken to improve data quality affecting the representativeness of subsequent research findings using the same case definitions.

### Accuracy of self-reported data for determining health outcomes

Self-reported outcome data are often viewed unfavorably compared with hospital-reported or physician-collected data. However, several studies considering the accuracy of self-reporting of VTEs in comparison with physician-collected data have found little to substantiate this view. Heckbert et al. (48) evaluated the agreement between self-reports and hospital discharge codes for 99,500 participant reports in the Women’s Health initiative. The concordance between self-reported and hospital-reported events was good (κ = 0.67 for PE and κ = 0.71 for DVT). However, both self-reported and hospital-reported events had higher concordance with physician-adjudicated events for PE (κ = 0.83 and κ = 0.84, respectively) and for DVT (κ = 0.72 and κ = 0.80) (48). These are much higher levels of agreement than we saw in the UK Biobank, which may be because participants were asked specifically about PE and DVT, whereas the UK Biobank asked an open question about physician-diagnosed conditions. Another possibility is that the low overlap reflects the fact that the self-reports referred mostly to events occurring prior to registration, while the bulk of the hospital data were collected after registration. Several much smaller studies have found similar concordances. Frezzato et al. (49) demonstrated that the question, “Do you think you ever had venous thromboembolism?” had a sensitivity of 84% and a specificity of 88% compared with medical records among 267 Italian participants. Greenbaum et al. (50) found an 88.9% positive predictive value for PE and a 69.7% positive predictive value for DVT when comparing self-reports with surgeons’ assessments in a US cohort of 3,976 postsurgery patients. This leads us to conclude that there is no strong inherent reason to disregard the self-reported data on VTEs as less accurate than the medical reports.

There is also a considerable body of literature on potential sources of bias in externally validated data. One concern is informed presence bias, which is influenced by socioeconomic factors, such as health-care costs (51), educational level, and time needed to travel to health-care services (52). Perceptions about the health-care system can affect patients’ willingness to self-report outcomes (53). Poor communication between patient and clinician could also be a factor in discordance, as more complicated conditions are both harder to diagnose (and thus underreported in medical records) and harder for the patient to understand (and thus misreported or underreported in self-reported data). This might explain why patients are more likely to self-report DVTs, a more commonly understood illness than PE. These factors mean that 2 patients with identical symptoms and underlying conditions may be recorded differently as VTE cases or controls in different data sources.

### Biases affecting VTE reporting

We found that changing the data source for defining a VTE outcome from hospital data to self-reported data altered the sociodemographic characteristics of cases under consideration. There was also noticeable variation between VTE case proportions by assessment center; this could have been due to underlying geographic variation in National Health Service provision, such as the availability of general practitioner appointments, the experience of local specialists, and the intensity of use of diagnostic scanning equipment (54). Self-reported cases were younger and mostly female, while hospital-defined cases were mostly male. This is particularly salient given conflicting evidence on whether VTEs are more prevalent in male or female patients and the impact this perception might have on subsequent diagnoses.

Several different factors might explain why women are more likely to self-report a disease without an equivalent medical record. Previous investigations have suggested age as a potential reason for differences in case prevalence, and self-reported events are mostly captured prior to registration (2, 3, 20). Prevalent events are subject to survivorship bias, but it is unclear why this would induce a gendered difference. We observed a difference in the mean age of cases between self-reported and hospital data; the absolute difference was 0.6 years. However, this difference is unlikely to explain such a large discrepancy in sex rates. It is also possible that this discrepancy is a result of sex bias in diagnosis. Diagnostic and treatment bias according to sociodemographic factors is well-documented for cardiovascular diseases. Worldwide, women are less likely to undergo a detailed risk factor assessment for cardiovascular disease even when physicians are presented with identical symptoms (55, 56) and are more likely to be misdiagnosed (57, 58) or to have their symptoms dismissed as psychogenic (59). Women with cardiovascular symptoms are less likely than men to be referred to a specialist (60, 61) and to receive advanced diagnostics (62), coronary procedures (63, 64), and appropriate drug treatment (65–67). It is unclear to what extent this generalizes specifically to VTEs: One study of DVT events found more women than men were sent for a diagnostic workup for DVT, but the actual diagnosis of DVT was higher in men with more severe thrombotic events (68). However, women have poorer quality-of-life outcomes 1 year after diagnosis (69), worse bleeding outcomes, and more VTE mortality in long-term follow-up (70). Given this, the magnitude of the impact of sex bias on VTE reporting is uncertain.

### Strengths and weaknesses of study

The strengths of our study include the large sample size. The previously largest study comparing self-reported VTEs with hospital reports was the Women’s Health Initiative. This study was one-fifth the size of the UK Biobank study, and only outcomes in women were considered (48). In other comparisons, study populations have been much smaller (22, 49, 71) or investigators have evaluated a more general cardiovascular outcome (72–75). Our results are also strengthened by the robust data linkage between the self-reported and hospital data. We know that the same participants appeared in both data sets; thus, differences in prevalence were not due to the biases of different samples but due to how well the different sources captured case numbers. Use of the linked primary-care data allowed us to investigate whether primary-care diagnosis could explain why so many participants only self-report VTE events. A further strength is the comparison of DVT and PE events. Because these events are caused by related biological mechanisms, differences in diagnosis and reporting patterns between the diseases will more strongly represent differences arising from social, behavioral, and clinical factors.

Weaknesses of our study include the fact that the UK Biobank is not representative of the UK population. This nonrepresentativeness limits our ability to extend conclusions beyond this data set, and none of the figures given here should be used as accurate estimates of the prevalence of VTE in the UK population. Nevertheless, the UK Biobank has a large influence on health research and perceptions about medical conditions worldwide. As such, it is vital to identify potential biases that may be introduced in considering particular data sources within the UK Biobank.

Another weakness is that the choices made in defining our primary-care cohort may have introduced additional bias. The primary-care cohort appears to be reasonably representative of the UK Biobank population in most characteristics but is distributed differently geographically. We acknowledge both the reproduction of the original biases of the UK Biobank and the possible intensification of them.

It is unclear whether these patterns found for VTEs in the UK Biobank would be similar for other conditions. Studies have found that more widely recognized and easily diagnosed illnesses tend to have greater agreement between self-reports and official records (74, 75), that community-managed conditions are less likely to be reflected in hospital records (73), and that more serious diseases have higher agreement between sources (76). However, there is not much consistency in how accuracy is reported between these studies, and it is difficult to form a conclusion about whether a specific disease will have a strong overlap between hospital records and self-report. We would expect, in line with the previous literature, that these differences will be less marked for more common and more well-known diseases, and for diseases for which there are empirical tests for diagnosis; this is reflected in our findings for DVT and PE.

### Recommendations

For studying VTEs and in general, we recommend that researchers look at the reports coming from all the possible sources within the UK Biobank, how the reports overlap, and whether there are any clues in the medication or demographic data that may help them identify the most appropriate definition to use. Self-reported data are particularly useful for identifying cases before baseline, while hospital data will capture more incident events. Inclusion of primary-care data may result in an analysis with less powered analysis due to the smaller number of participants with available data, but it has the potential to capture events rarely seen in hospital, such as depression. While primary-care data were not useful in validating self-reported events in the case of VTEs, there may be conditions where there is a much larger overlap between sources of report, in which case the self-reported data could be used as a proxy for the missing primary-care data in the full cohort. We would recommend that self-reported data be included for case definitions of VTE, either as sensitivity analysis alongside a more parsimonious main definition or as the primary analysis together with a sensitivity analysis that excludes the self-report data. This gives the researcher the greatest flexibility for understanding the impact this decision might have on their analysis.

In conclusion, there are large differences between the VTE case populations defined based on routinely collected hospital data and those defined based on self-reported data in the UK Biobank, in terms of both the number of events reported and the demographic characteristics of the case populations. Such differences are likely to affect our perception of the typical VTE patient. As such, our findings suggest that in future studies, researchers need to be aware of potential demographic differences underlying seemingly neutral event definitions in order to avoid entrenching further inequalities in health care.

## Supplementary Material

Web_Material_kwad232
