# Inconsistency in UK Biobank event definitions from different data sources and its impact on bias and generalizability: a case study of venous thromboembolism

**DOI:** 10.1093/aje/kwad232

**Published:** 2023-11-17

**Authors:** Emily Bassett, James Broadbent, Dipender Gill, Stephen Burgess, Amy M. Mason

**Affiliations:** MRC Biostatistics Unit, University of Cambridge, Cambridge, UK; MRC Biostatistics Unit, University of Cambridge, Cambridge, UK; York University, Toronto, Canada; Department of Epidemiology and Biostatistics, School of Public Health, Imperial College London, UK; Chief Scientific Advisor Office, Research and Early Development, Novo Nordisk, Copenhagen, Denmark; MRC Biostatistics Unit, University of Cambridge, Cambridge, UK; British Heart Foundation Cardiovascular Epidemiology Unit, Department of Public Health and Primary Care, University of Cambridge, Cambridge, UK; Victor Phillip Dahdaleh Heart and Lung Research Institute, University of Cambridge, Cambridge UK; MRC Biostatistics Unit, University of Cambridge, Cambridge, UK; British Heart Foundation Cardiovascular Epidemiology Unit, Department of Public Health and Primary Care, University of Cambridge, Cambridge, UK; Victor Phillip Dahdaleh Heart and Lung Research Institute, University of Cambridge, Cambridge UK

**Keywords:** UK Biobank, Bias, Generalizability, Representativeness, Venous Thromboembolism, Event definition, Sociodemographic characteristics, Deep vein thrombosis, Pulmonary embolism

## Abstract

The UK Biobank study contains several sources of diagnostic data, including hospital inpatient data and self-reported conditions for ~500,000 participants, and primary care data for ~177,000 participants (35%). Epidemiological investigations require a primary disease definition, but whether to combine sources to maximize power or focus on one to ensure a consistent outcome is not clear. The consistency of definitions was investigated for venous thromboembolism (VTE) by looking at overlap when defining cases from hospital in-patient data, primary care reports, and self-reported questionnaires. VTE cases showed little overlap between data sources, with only 6% of reported events for those with primary care data identified by all three of hospital, primary care, and self-report, while 71% appeared only in one source. Deep vein thrombosis only events represented 68% of self-reported and 36% of hospital-reported VTE cases, while pulmonary embolism only events represented 20% of self-reported and 50% of hospital-reported VTE cases. Additionally, different distributions of sociodemographic characteristics were observed; for example, 46% of hospital reported VTE cases were female, compared with 58% of self-reported VTE cases. These results illustrate how seemingly neutral decisions taken to improve data quality can affect the representativeness of a dataset.

## Background

Venous thromboembolism (VTE) is a condition that occurs when a blood clot forms inside the veins, preventing blood flow. Its incidence is roughly 100 events per 100,000 person-years^1,2^. Approximately two-thirds of cases are deep vein thrombosis (DVT)^3–5^, where the blood clot forms in a deep vein, typically the pelvis, thigh, or lower leg. A third of cases are pulmonary embolism (PE), which occurs when the clot breaks loose and travels to the lungs^3–5^. In rare cases, thrombosis may occur in other veins.

Factors associated with greater risk of VTE include obesity^6^, height^7^, smoking status^8^, hypertension^9^, social deprivation^10,11^, education^9^, immobilisation^12^, surgery^13^, use of hormone replacement therapy (HRT) or oral contraceptives^14,15^, and pregnancy^16^. Risk of VTE also increases with age, as does the proportion of VTEs that are PEs^17^. There is little consistent evidence for overall differences in VTE risk by sex: there are reports of higher rates for men^2,9,17^, no significant difference^18,19^, and higher rates for women^3,20–22^ when combining across all ages. However, there may be different patterns of risk across lifetime, with risk in women higher in reproductive years and risk in men higher in old age^2,3,20^, and men at higher risk of recurrent events^23,24^. VTE risk is higher for individuals of African ancestry than European ancestry^25^, and higher for individuals of European ancestry than Hispanic and Asian ancestry^26,27^.

Previous studies on VTE in UK Biobank have used a combination of self-reported physician diagnosis of DVT or PE, details from hospital inpatient records, and death certificates, or a subset of these sources^28–30^. Details from primary care records in UK Biobank are less often used as they are not available for the entire cohort. Other studies have used different sources to determine VTE cases. A 23andMe study used self-reported VTEs alone^31^, while a large Norwegian study used a combination of inpatient and outpatient hospital records^3^. As studies do not typically breakdown results by source of diagnosis report, it is unclear how much different sources of report could impact the number and sociodemographic makeup of identified cases.

Electronic health records used for research are susceptible to “informed presence bias”. Patients do not appear in health records at random, but are influenced by the symptoms they have and how well they can communicate them to clinicians^32,33^. Clinician suspicion determines who is scored for suspected DVT/PE. A probability assessment using the modified deep vein thrombosis and pulmonary embolism Wells Scores determines who then gets to access further tests such as D-dimer measurement, ultrasound, and radiological imaging^34^. Currently in the UK’s National Health Service, individuals with DVT and low-risk PE can be treated as outpatients^35^, while those with high-risk PE would be admitted as inpatients – this could influence which data sources record a VTE and whose VTEs get recorded.

The aim of this investigation is to determine how using different sources of data may impact VTE case populations within UK Biobank. We will do this by considering how closely reports of VTE from different data sources correspond and whether the populations reported as cases are similar. We will not consider any specific reporting method as a “gold standard” of truth to determine the accuracy of other methods, nor will we attempt to estimate VTE incidence in the general UK population. Instead, we will compare how similar each definition is to the others within UK Biobank.

## Methods

### Study participants

The UK Biobank is a large prospective cohort study containing diagnostic data for 503,317 participants, aged 37 to 73 years, recruited across England, Scotland, and Wales between 2006 and 2010.

### Data sources within UK Biobank

#### Self-reported outcomes

At enrolment and resurvey, participants answered a touch-screen questionnaire, including specific questions about prior physician diagnoses of blood clots in the leg or lungs as well as more general questions about serious medical conditions. These were followed up with a verbal interview^36^. Where participants were not certain about prior diagnoses, their responses were matched where possible to health conditions in a coding tree by a medical professional^37^. Self-reported VTEs were coded as either DVT, PE, or other VTE.

#### Hospital data

ICD-9 and -10 coded hospital inpatient episodes were obtained from the Hospital Episode Statistics provider for England, the Patient Episode Data for Wales, and the Scottish Morbidity Records for Scotland^38^. These datasets contain information on admission and discharge, operations, diagnoses, maternity care, and psychiatric care. Main and secondary diagnoses throughout the patient admission are recorded. This data is only available within UK Biobank for patients who are admitted to the hospital and occupy a bed.

#### Death certificate data

ICD-10 coded national death registry data were obtained from the Health and Social Care Information Centre (now NHS England) for England and Wales, and the Information Services Department (ISD) for Scotland^39^. This includes primary and secondary causes of death determined by a doctor who attended the patient in their last illness or a coroner^40^.

#### Primary care records

Primary care data were captured for 230,000 participants, covering records from selected general practice services in England, Scotland, and Wales^41^. We took a subset of 177,363 participants that ensured continuous coverage overlapping with their recruitment into UK Biobank – details on choices made can be found in Web Appendix 1.

### Event definitions

VTE cases were determined using the four data sources: death certificate data, hospital data, self-reported outcomes, and primary care records (in the primary care cohort only). We considered events reported by each data source in turn, and a combined outcome including events reported by any data source.

VTE cases were broken down into PE and DVT via matching to any of the codes in Web Table 1. If a participant matched to VTE, but not PE or DVT they were classed as “Other VTE”.

#### Medication use

One concern about the self-reported outcomes is that case definitions may be much less accurate. To investigate this concern, we considered whether patterns of relevant medication use were similar between the cases reported via different sources.

VTEs are often treated with anti-coagulants. While warfarin is not recommended as the first line treatment in the current UK guidelines, the standard of care prior to 2020 was a low molecular-weight heparin bridge followed by warfarin^34^.

There are two sources of general medication usage data within UK Biobank. One is self-reported, collecting lists of all regularly taken prescription medications during the touch screen questionnaire and verbal interview (data-field 20003)^36^. The other is the primary care records prescription data, which is only available for the cohort with primary care data (data-field 42039). Matching on drug names was undertaken to identify participants who had taken either any anticoagulant or warfarin at some point (details of matching in Web Table 2)

### Statistical methods

We cross-tabulated the events in both the full UK Biobank sample and in the cohort with available primary care data. In both groups, we compared anti-coagulant medication use and demographic data defined by the various data sources. The variables we compared were age at baseline, gender, smoking status, ethnicity, body mass index (BMI), current employment status, highest level of education, history of manual or shift work, Townsend deprivation index (greater means more deprived), house ownership, and car ownership. For participants in England, we also looked at the Index of Multiple Deprivation (IMD) and the scores that determine the IMD.

Proportional Venn diagrams were plotted to get a visual understanding of the various overlaps of cases. Agreement between the methods was evaluated using Fleiss Kappa between all the sets and Cohan Kappa pairwise between each method. Kappa coefficients <0.6 are taken as inadequate agreement in line with recommendations for health-related studies, 0.6-0.8 as moderate agreement, 0.8-0.9 as strong agreement and >0.9 as almost perfect agreement^42^.

## Results

### Study population

Table 1 contains a summary of the overall demographics of UK Biobank. The defined primary care cohort reproduces the known biases within the UK Biobank dataset – there is a “healthy volunteer bias” with participants more likely to be older, female, have a lower BMI, smoke less, live in less socioeconomically deprived areas, and have a greater rate of higher education than the average person in the UK^43^ (Web Table 3). The primary care cohort has a similar gender and age balance, a slightly greater proportion of White participants, higher rates of unemployment, and lower rates of higher education compared with the full dataset.

### Event definitions in full UK Biobank sample

No single data source captures all VTE cases, and the percentage captured by different methods varies by case type. Taking a report from any source as a case and breaking down by sub diagnosis: 13% had both PE and DVT, 54% had DVT only, 30% had PE only and 3% had a VTE that fit into neither category.

**Figure 1:**
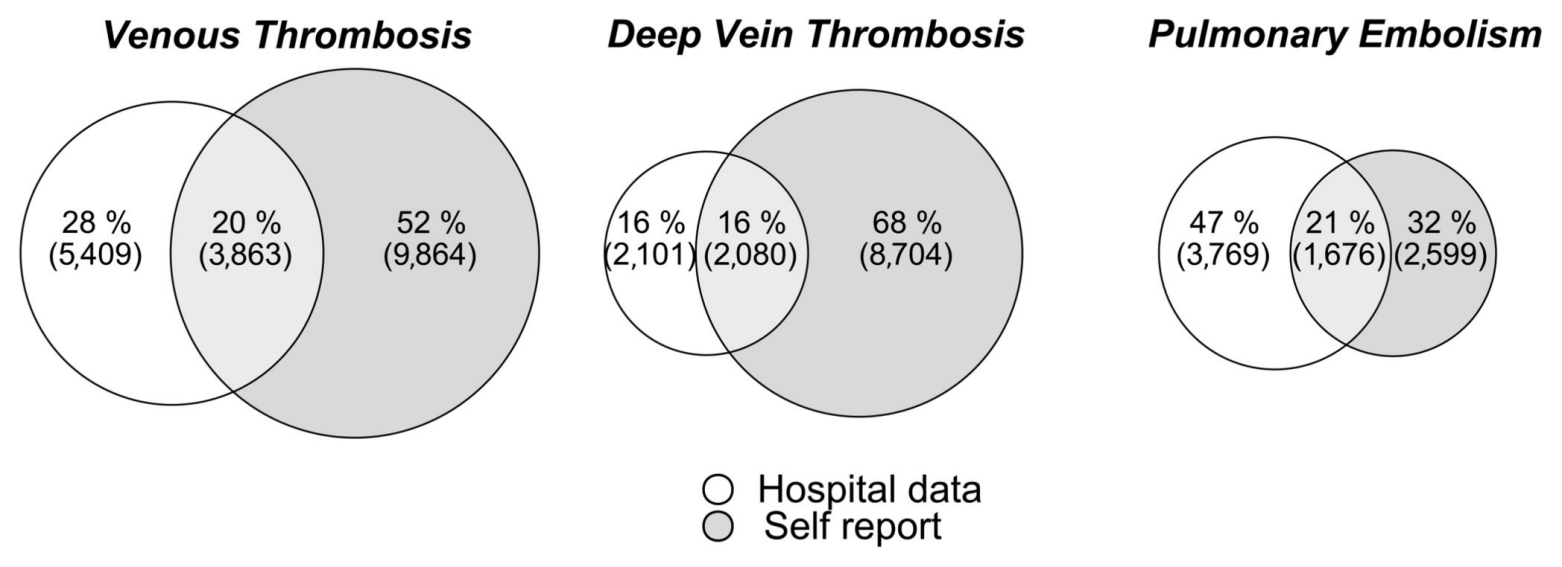
Venn diagram of the proportional overlap in VTE cases between different data sources in all UK Biobank participants. These are expanded further into events prior to and post registration in UK Biobank in Web Figure 1.

There is little agreement between self-report and inpatient hospital data (κ = 0.32). Only 20.2% of VTE cases are reported by both sources (Figure 1), while 79.8% appear only in a single source (51.5% appear only in self-reports and 28.3% appear only in hospital data). There is a larger overlap of hospital events being self-reported when we restrict to prevalent events, but we do not see a matching hospital report for the majority of incident self-reported events (Web Figure 1).

The data from death certificates did not add any additional clarity (Web Tables 4 and 5, Web Figures 2 and 3). There were 741 cases of VTE identified from primary and secondary causes of death, of which 388 cases did not appear in another data source. Due to the small proportion of cases identified through this method (~4%), we did not analyse death certificate data further.

Considering the two sub-diagnoses (DVT and PE), there is a difference in the source of the report by sub diagnosis: more DVTs are only self-reported (67.6%) than are in hospital records only (16.3%) or both in the hospital records and self-reported (16.1%), whereas PE are most likely to be in hospital records only (46.9%) although nearly a third appear only as self-reports (32.3%).

**Figure 2:**
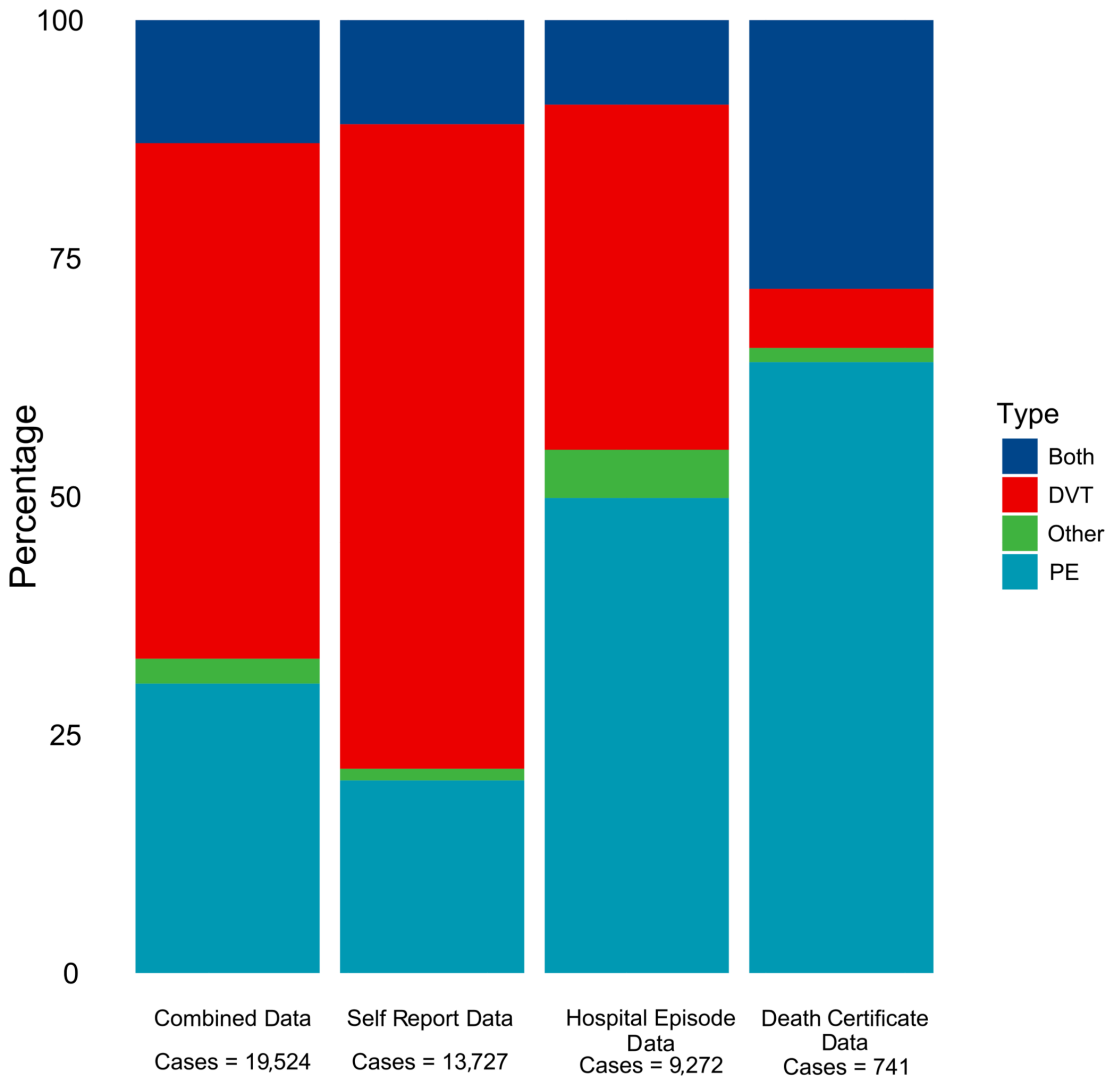
Proportions of Deep Vein Thrombosis (DVT) to Pulmonary Embolism (PE) in identified venous thromboembolism (VTE) cases when ascertaining cases from different sources.

The proportion of DVT to PE events also varies with the data source (Figure 2). If we consider only hospital data, 50% of events are PE only, 36% DVT only, and 9% both; whereas taking self-reported outcomes as the data source, 20% of events are PE only, 68% DVT only, and 11% are both.

We also see variation in the demographics of the identified cases (Table 2). For example, using only hospital data the case population is 45.7% female, while using the self-reported data the case population is 58.3% female.

### Event definitions in primary care cohort

**Figure 3:**
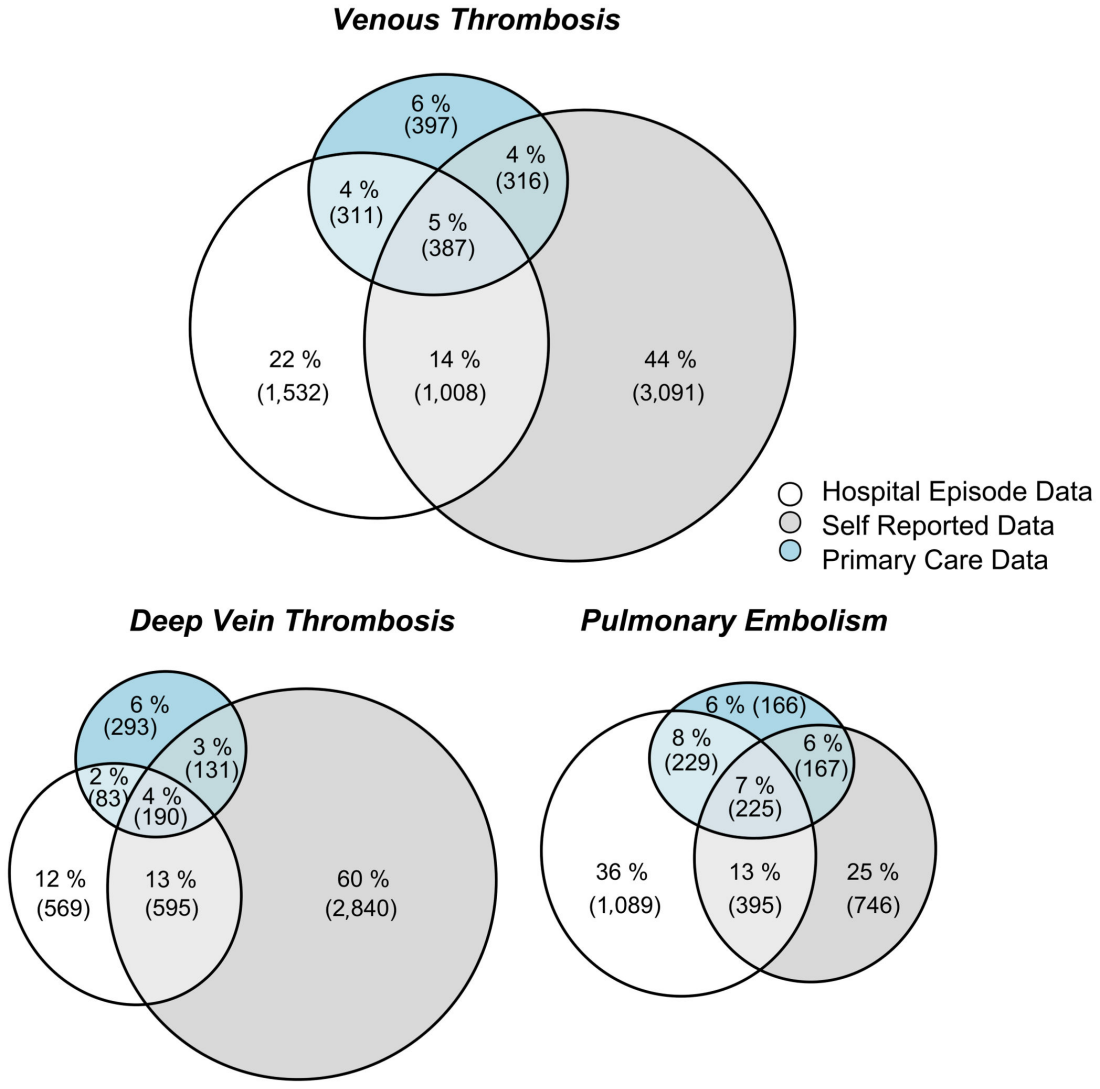
Venn diagram of the proportional overlap in VTE cases between different data sources in the UK Biobank participants with primary care data. These are expanded further into events prior to and post registration in UK Biobank in Web Figures 4 & 5.

The primary care cohort within UK Biobank shows similar patterns of case overlap to the full participant group (Figure 3). Adding the additional cases from the primary care records does not explain many of the undocumented self-reported VTE events and adds an additional set of otherwise uncaptured outcomes.

The highest agreement is between hospital and self-reported data (κ = 0.33), but this is still inadequate in terms of concordance. Primary care data have slightly more agreement with hospital data than self-reported data (κ = 0.29 vs 0.21). Only 5.5% of VTE cases are reported by all three sources, while 71.3% appear only in a single source: 43.9% appear only in self-reports, 21.8% appear only in hospital data, and 5.6% appear only in primary care reports. Splitting into prior and post registration, there is a clear time-period effect due to the lack of self-reports post registration for many participants and the sparsity of hospital reports prior to registration. In all cases, the primary care data and the hospital data have little overlap. (Web Figure 4 and Web Figure 5; Web Tables 6-8).

There is a difference in the source of the report for the sub diagnoses: most DVTs are only self-reported (60.4%), while more PEs are in hospital records only (36.1%) than in any other category. There is slightly better agreement between sources for PE (κ between 0.33-0.35 when comparing hospital data, primary care data, and self-report) than for DVT (κ between 0.14-0.27). See Web Table 9.

### Anticoagulant usage in the primary care cohort

There are different pattens of reported anticoagulant use between the different case groups, but all have much higher rates than the control group (Web Table 10). Cases of VTE identified using only hospital data are more likely to have a record of anticoagulant drug use at some point in their primary care records (64.7% used some sort of anticoagulant, 50.4% were on warfarin) whereas those identified via primary care records only had much lower use (37.9% and 26.8% respectively). Self-reported only cases fell between these two groups (51.2% and 33.2% respectively). In contrast, anticoagulant drug use amongst controls (that is, individuals with no reported VTE event from any source) was much lower (18.9% and 2.5% respectively). This provides indication that there are likely to be true VTE events amongst the self-reported only cases. Self-reported rates of anticoagulant use were much lower, but more consistent between definitions (Web Figure 6).

### Differences in socio-demographics between cases from each data source

The self-reported cases are younger and more likely to be female than the hospital data cases. They are more likely to have been assessed at the UK Biobank centres in Wales, and less likely to have been assessed in Scotland. There are also differences between these two case groups in terms of mean BMI, house ownership, and multiple car ownership. The cases identified by primary care data are somewhere between the other two case groups in terms of both gender and age, with lower levels of deprivation, and higher rates of house and multiple car ownership.

## Discussion

Our investigation found using different data sources in UK Biobank results in substantial differences in the number, balance, and socio-demographic characteristics of VTE cases considered. None of the data sources have good agreement with each other. The majority of DVT events appear only as self-reported outcomes. For PE, the largest group of events is reports from hospital data only. One likely reason for this is severity, with DVTs more likely to be treated in outpatient settings^35^ while PE is more often life-threatening, resulting in hospitalization. Hospital reports constitute the majority of post-registration events in the study, while the majority of events prior to registration are self-reported. However, this is not a pure effect of time-period, as there are self-reports after registration that are not seen in hospital records and hospital reports before registration than are not-self reported. For both diseases, only a small proportion of participants are detected by multiple data sources as having an event. This suggests a need to be attentive to how use of different data sources may influence case definition and composition.

Large studies into patient characteristics affect our perception of diseases: for example, studies claiming VTE predominantly affects male or female patients likely impact physicians’ perception of reported symptoms, as has been seen for cardiovascular disease^44^ and depression^45^. This can impact how readily they diagnose future patients. As a result, decisions drawn from biased data can lead to greater health inequality, as has previously been observed for algorithmic decisions^46,47^. Future studies can also be biased by these perceptions with well-meaning and seemingly neutral decisions taken to improve data quality impacting the representativeness of subsequent research findings using the same case definitions.

### Accuracy of self-reported data for determining health outcomes

Self-reported outcome data are often viewed unfavourably compared to hospital-reported or physician-collected data. However, several studies considering the accuracy of self-reporting of VTEs compared to physician collected data have found little to substantiate this view. Heckbert et al. looked at the agreement between self-report and hospital discharge codes for 99,500 participant reports in the Women’s Health initiative. The concordance between self-reported and hospital-reported events was good (κ = 0.67 for PE, 0.71 for DVT). However, both self-reported and hospital reported events had higher concordance with physician-adjudicated events for PE (κ = 0.83 and 0.84 respectively), and for DVT (κ = 0.72 and 0.80)^48^. This is much higher levels of agreement than we saw in UK Biobank, which may be because participants were asked specifically about PE and DVT, whereas UK Biobank asked an open question about physician diagnosed conditions. Another possibility is that that the low overlap is because the self-reports mostly refer to events prior to registration, while the bulk of the hospital data is after registration. Several much smaller studies have found similar concordances. Frezzato et al. showed the question “Do you think you ever had venous thromboembolism?” had a sensitivity of 84% and specificity of 88% compared to medical records in 267 Italian participants^49^. Greenbaum et al. found 88.9% positive predictive value for PE and 69.7% positive predictive value for DVT comparing self-report with surgeon assessment in a US cohort of 3976 post-surgery patients^50^. This leads us to conclude that there is no strong inherent reason to disregard the self-reported data on VTEs as less accurate than the medical reports.

There is also considerable literature on potential sources of bias in externally validated data. One concern is informed presence bias, which is influenced by socioeconomic factors, such as healthcare costs^51^, levels of education and distance to travel to healthcare^52^. Perceptions about the healthcare system can impact patient willingness to self-report outcomes^53^. Poor communication between patient and clinician could also be a factor in discordance, as more complicated conditions are both harder to diagnose (and thus underreported in medical records) and hard to understand for the patient (and thus mis-reported or underreported in self-reported data). This might explain why patients are more likely to self-report DVTs, a more commonly understood illness than PE. These factors mean that two patients with identical symptoms and underlying conditions may be represented in different data sources.

### Biases impacting VTE reporting

We found changing the data source for defining VTE outcome from hospital data to self-reported data altered the socio-demographic characteristics of cases under consideration. There is also noticeable variation between VTE case proportions by assessment centre - this could be due to underlying geographic variation in NHS provision.^54^ Self-reported cases were younger and majority female, while hospital-defined cases were majority male. This is particularly salient given conflicting evidence on whether VTEs are more prevalent in male or female patients and the impact this perception might have on subsequent diagnoses.

Several different factors might explain why women are more likely to self-report a disease without an equivalent medical record. Previous investigations have suggested age as a potential reason for differences in case prevalence, and self-reported events are mostly captured prior to registration ^2,3,20^. Prevalent events are subject to survivorship bias, but it is unclear why this would induce a gendered difference. We observed a difference in the mean age of cases between self-reported and hospital data, the absolute difference was 0.6 years. However, this difference is unlikely to explain such a large discrepancy in gender rates. It is also possible that this discrepancy is a result of gender bias in diagnosis. Diagnostic and treatment bias on sociodemographic factors is well documented for cardiovascular diseases. Worldwide, women are less likely to undergo a detailed risk factor assessment for cardiovascular disease even when doctors are presented with identical symptoms^55,56^ and more likely to be misdiagnosed^57,58^ or have their symptoms dismissed as psychogenic^59^. Women with cardiovascular symptoms are less likely than men to be referred to a specialist^60,61^, receive advanced diagnostics^62^, coronary procedures^63,64^ and appropriate drug treatment^65–67^. It is unclear to what extent this generalises specifically to VTEs: a study of DVT events found more women than men were sent for a diagnostic workup for DVT, but the actual diagnosis of DVT was higher in men with more severe thrombotic events^68^. However, women have poorer quality of life outcomes 1 year after diagnosis^69^, worse bleeding outcomes, and more VTE mortality in long term follow up^70^. Given this, the magnitude of the impact of gender bias on VTE reporting is uncertain.

### Strengths and weaknesses of study

The strengths of our study include the large sample size. The previous largest study comparing self-reported VTEs to hospital reports used the Women’s Health Initiative study. This was one-fifth the size of UK Biobank, and only considered outcomes in women^48^. Other comparisons have either been much smaller^22,49,71^, or looked at a more general cardiovascular outcome ^72–75^. Our results are also strengthened by the robust data linkage between the self-reported and hospital data. We know that the same participants appear in both datasets, thus differences in prevalence are not due to the biases of different samples but due to how well the different sources capture case numbers. Use of the linked primary care data allowed us to investigate whether primary care diagnosis could explain why so many participants only self-report VTE events. A further strength is the comparison of DVT and PE events. As these events are caused by related biological mechanisms, differences in diagnosis and reporting patterns between the diseases will more strongly represent differences arising from social, behavioural, and clinical factors.

Weaknesses in our study include the fact that UK Biobank is not representative of the UK population. This non-representativeness limits our ability to extend conclusions beyond this dataset and none of the figures here should be used as accurate estimates of the prevalence of VTEs in the UK population. Nevertheless, the UK Biobank has a large influence on health research and perceptions about medical conditions worldwide. As such, it is vital to identify potential biases that may be introduced in considering particular data sources within UK Biobank.

Another weakness is that the choices made in defining our primary care cohort may introduce additional bias. The primary care cohort appears to be reasonably representative of the UK Biobank population in most characteristics but is distributed differently geographically. We acknowledge both the reproduction of the original biases of UK Biobank and the possible intensification of them.

It is unclear whether these patterns found for VTEs in UK Biobank would be similar for other conditions. Studies have found that more widely recognised and easily diagnosed illnesses tend to have greater agreement between self-report and official records ^74 75^, that community managed conditions are less likely to be reflected in hospital records^73^ and that more serious diseases have higher agreement between sources^76^. However, there is not much consistency in how accuracy is reported between these studies and it is difficult to conclude whether a specific disease will have a strong overlap between hospital records and self-report. We would expect, in line with the previous literature, that these differences will be less marked for more common and more well-known diseases, and for diseases where there are empirical tests for diagnosis; this is reflected in our findings for DVT and PE.

### Recommendations

For studying VTEs, and in general, we recommend that researchers look at the reports coming from all the possible sources within UK Biobank, how the reports overlap, and whether there are any clues in the medication or demographic data that may help them identify the most appropriate definition to use. Using self-reported data is particularly useful for identifying cases before baseline, while hospital data will capture more incident events. Including primary care data may create a lower powered analysis due to the smaller number of participants with available data, but it has the potential to capture events rarely seen in hospital, such as depression. While primary care data were not useful in validating self-reported events in the case of VTEs, there may be conditions where there is a much larger overlap between sources of report, in which case the self-reported data could be used as a proxy for the missing primary care data in the full cohort. We would recommend that self-reported data be included for case definitions of VTE, either as sensitivity analysis alongside a more parsimonious main definition or as the primary analysis together with a sensitivity analysis that excludes the self-report data. This gives the researcher the greatest flexibility for understanding the impact this decision has on their analysis.

In conclusion, there are large differences between the VTE case populations defined based on routinely collected hospital data and based on self-reported data in UK Biobank, both in terms of the number of events reported and the demographics of the case populations. Such differences are likely to affect our perception of the typical VTE patient. As such, our findings suggest that future studies need to take be aware of potential demographic differences underlying seemingly neutral event definitions in order to avoid entrenching further inequalities in healthcare.

## Supplementary Material

Supplementary material

## Figures and Tables

**Table 1:** Demographics of UK Biobank participants at recruitment

	All participants (n =502,520)		Primary care cohort (n= 177,358)	
	%	Mean (SD)	%	Mean (SD)
Female	54.4		54.5	
Age in years		57.0 (8.1)		57.2 (8.0)
White Ethnicity	94.6		95.7	
Wales Assessment Center	4.1		10.3	
Scotland Assessment Centre	7.1		12.6	
London Assessment Centre	13.7		6.8	
Unemployment	43.1		44.1	
Manual work ^a^	7.7		7.9	
Higher Education	60.2		59.8	
Townsend deprivation index		-1.29 (3.1)		-1.4 (3.0)
Own house outright	51.5		52.9	
Two or more cars in household	48.8		48.3	
Shift-working^b^	5.6		5.6	
Mean female BMI ^c^		27.1 (5.2)		27.2 (5.2)
Mean male BMI ^c^		27.8 (4.2)		27.9 (4.3)
Current smokers (Women)	8.9		8.7	
Current smokers (men)	12.5		12.1	

a Answered “Usually” or “Always” to “Does your work involve heavy manual or physical work?”

b Answered “Usually” or “Always” to “Does your work involve shift work?”

c Weight (kg)/height (m)^2^

**Table 2:** Demographic comparison between case populations defined via the different data sources. A larger version of this table can be seen as Web Table 11. P-values are reported for independent sample t-tests for continuous variables and chi-squared tests of proportions for binary variables.

Data source	Hospital data (n =9272)		Primary care data (n= 1441)		Self-reported (n =13,727)		P-value for difference between self-reported and hospital data
	%	Mean (SD)	%	Mean (SD)	%	Mean (SD)	
Female	45.7		54.0		58.3		< 0.0001
Age in years		60.3 (7.2)		60.1 (7.2)		59.7 (7.4)	< 0.0001
White ethnicity	96.5		97.3		96.0		0.051
Wales Assessment Centre	3.9		14.1		4.9		0.0003
Scotland Assessment Centre	7.0		11.7		5.8		0.0002
London Assessment Centre	11.4		5.3		11.7		0.486
Not working (retired or unemployed)	60.4		61.1		59.9		0.448
Higher Education	52.7		52.4		53		0.655
Current smokers	12.2		12.3		12.4		0.652
Heavy manual work history^a^	6.5		6.4		5.9		0.063
Shift work history^b^	4.6		4.4		4.5		0.721
Townsend deprivation index		-0.89 (3.31)		-1.07 (3.17)		-0.93 (3.27)	0.365
Own house outright	56.8		57.5		55.0		0.007
More than 1 car in household	40.7		41.2		42.4		0.010
BMI^c^		29.4 (5.6)		29.2 (5.6)		29.2 (5.7)	0.009

a Answered “Usually” or “Always” to “Does your work involve heavy manual or physical work?”

b Answered “Usually” or “Always” to “Does your work involve shift work?”

c Weight (kg)/height (m)^2^

## Data Availability

This research was conducted using the UK Biobank Resource under Application Number 20480. Individual level data from UK Biobank cannot be shared publicly for ethical & privacy reasons. The data will be shared on reasonable request to the corresponding author, with the permission of UK Biobank.
