# Supplementary material for: Inconsistency in UK Biobank event definitions from different data sources and its impact on bias and generalizability: a case study of venous thromboembolism
Source: Am J Epidemiol. Author manuscript; Available in PMC 2024 May 9. (PMC11074710; doi:10.1093/aje/kwad232)
Supplement: Supplementary material [file EMS192690-supplement-Supplementary_material.docx]

# Web material for Inconsistency in UK Biobank event definitions from different data sources and its impact on representativeness: a case study of venous thromboembolism.

Emily Bassett, James Broadbent, Dipender Gill, Stephen Burgess, Amy M. Mason

Web Appendix 1: Details on record selection for primary care data 2

[Web Figure 1 Venn diagram of the proportional overlap in VTE, DVT and PE cases between different data sources split into events prior to registration and events post registration. 3](#_Toc149920685)

[Web Figure 2 Venn diagram of the proportional overlap in DVT and PE cases between different data sources restricted to the primary care cohort in UK Biobank.. 4](#_Toc149920686)

[Web Figure 3 Venn diagram of the proportional overlap in VTE cases between different data sources restricted to the primary care cohort in UK Biobank including death certificate data. 5](#_Toc149920687)

[Web Figure 4 Venn diagram of the proportional overlap in VTE, DVT and PE cases in the primary care cohort from the difference reporting sources, split into events prior to registration and events post registration. 6](#_Toc149920688)

[Web Figure 5 Venn diagram of the proportional overlap in VTE, DVT and PE cases in the primary care cohort from the difference reporting sources, including death certificate split into events prior to registration and events post registration. Categories with <1% of events in have not been labelled for clarity. 7](#_Toc149920689)

[Web Figure 6 Difference in percentages of VTE cases reporting usage of general anticoagulants or warfarin split. 8](#_Toc149920690)

[Web Table 1 Definitions of Venous thromboembolism (VTE), Pulmonary embolism (PE) and Deep venous thrombosis (DVT) 9](#_Toc149925533)

[Web Table 2 List of names of medications marched with GP and self-report data, together with counts of each match found. The list of anticoagulants searched for were :Warfarin, Heparin, Indandione, Dalteparin, Coumadin, K-vitamin antagonist, Acenocoumarol and Aspirin 10](#_Toc149925534)

[Web Table 3 Comparison of UK Biobank demographics with general UK population 23](#_Toc149925535)

[Web Table 4 Count of cases in UK Biobank broken down by source(s) of report 24](#_Toc149925536)

[Web Table 5 Count of cases in the Primary Care cohort broken down by source(s) of report 25](#_Toc149925537)

[Web Table 6 Count of hospital and self-report cases in UK Biobank broken down by source(s) and timing of report 26](#_Toc149925538)

[Web Table 7 Count of cases in the Primary Care cohort broken down by source(s) of report, excluding information from death certificates 27](#_Toc149925539)

[Web Table 8 : Count of incident cases in the Primary Care cohort broken down by source(s) of report including information from death certificates. 28](#_Toc149925540)

[Web Table 9 Kappa Concordance statistics from different data sources for VTE and subdefinition. 29](#_Toc149925541)

[Web Table 10 Concordance of reports of medication use between self-reports and GP records for all anti-coagulants and warfarin; Percentages of participants on anticoagulant medication and warfarin by source of case definition 30](#_Toc149925542)

[Web Table 11 Demographic comparison between VTE case populations defined via the different data sources. 31](#_Toc149925543)

## Web Appendix 1: Details on record selection for primary care data

We attempted to create a primary care data set that is representative of UK Biobank. Primary services were selected based on whether they agreed to supply data on patients in their practise to UK Biobank, resulting in variable coverage. Due to issues of reporting bias with data supplied by Vision in England, we restricted comparison to data from Scotland and Wales, and data supplied by TPP in England. Vision only supplied data for people still alive at date of extraction, which was 7-13 years after registration with UK Biobank. We further restricted to ensure continuous coverage: dropping records that ended before start of study (2696 people), started after the current censor date on the record from UKB (1699 people) or that started outside of 1904 to 2030 (14,434 people). We also required that there be at least one record before the start of the study (34,125 participants removed). Records were censored either at the first gap in registration of more than 1 week or at the UKB censoring dates^1^. This left a remaining cohort of 177,363 people with a continuous period of primary care data that overlaps with the recruitment date into UK Biobank.

1. UK Biobank. *UK Biobank Primary Care Linked Data Version 1.0*. 2019.


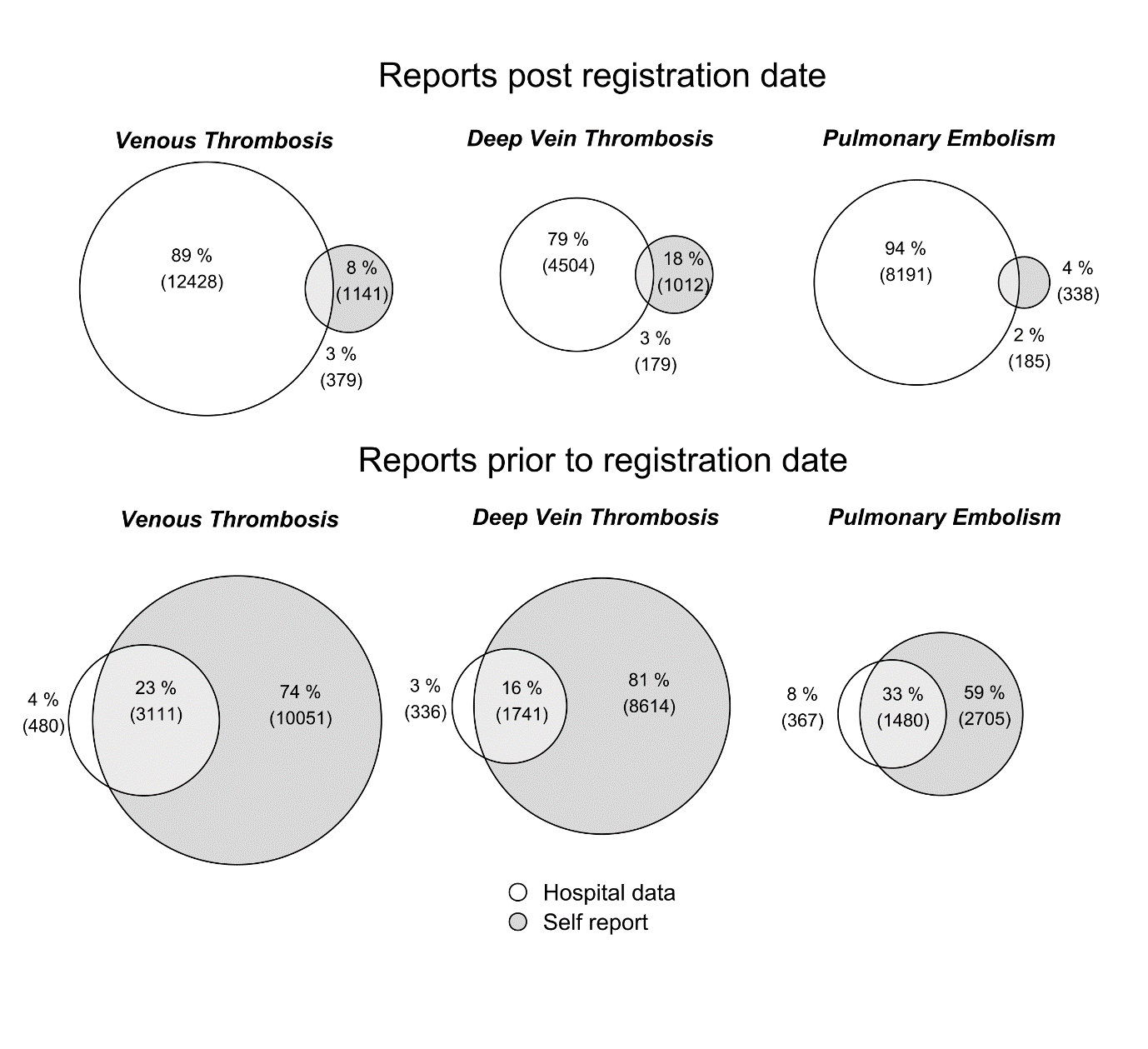


Web Figure 1 Venn diagram of the proportional overlap in VTE, DVT and PE cases between different data sources split into events prior to registration and events post registration. Death Certificate data was not included in this diagram. Self reported event post baseline were only available for a smaller proportion of UK Biobank who volunteered for follow up studies. See Web table 9 for exact numbers in these categories.


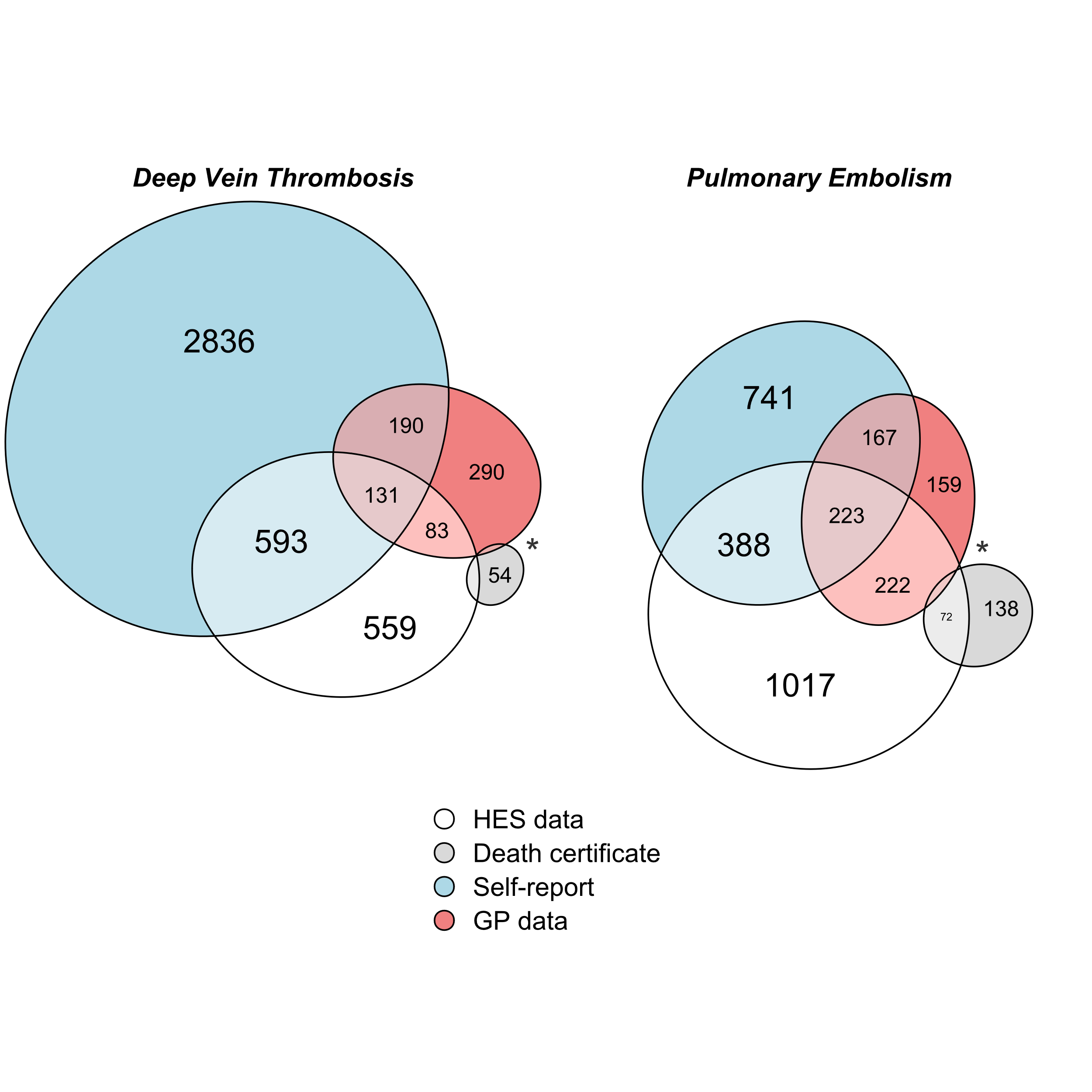


Web Figure 2 Venn diagram of the proportional overlap in DVT and PE cases between different data sources restricted to the primary care cohort in UK Biobank. Sets with <20 participants have not been labelled. The asterisk indicates that this diagram is not accurate for the overlap of death certificate data – there are very small numbers of overlap with the self-report data that are not possible to display. See Web table 5 for exact numbers in these categories.


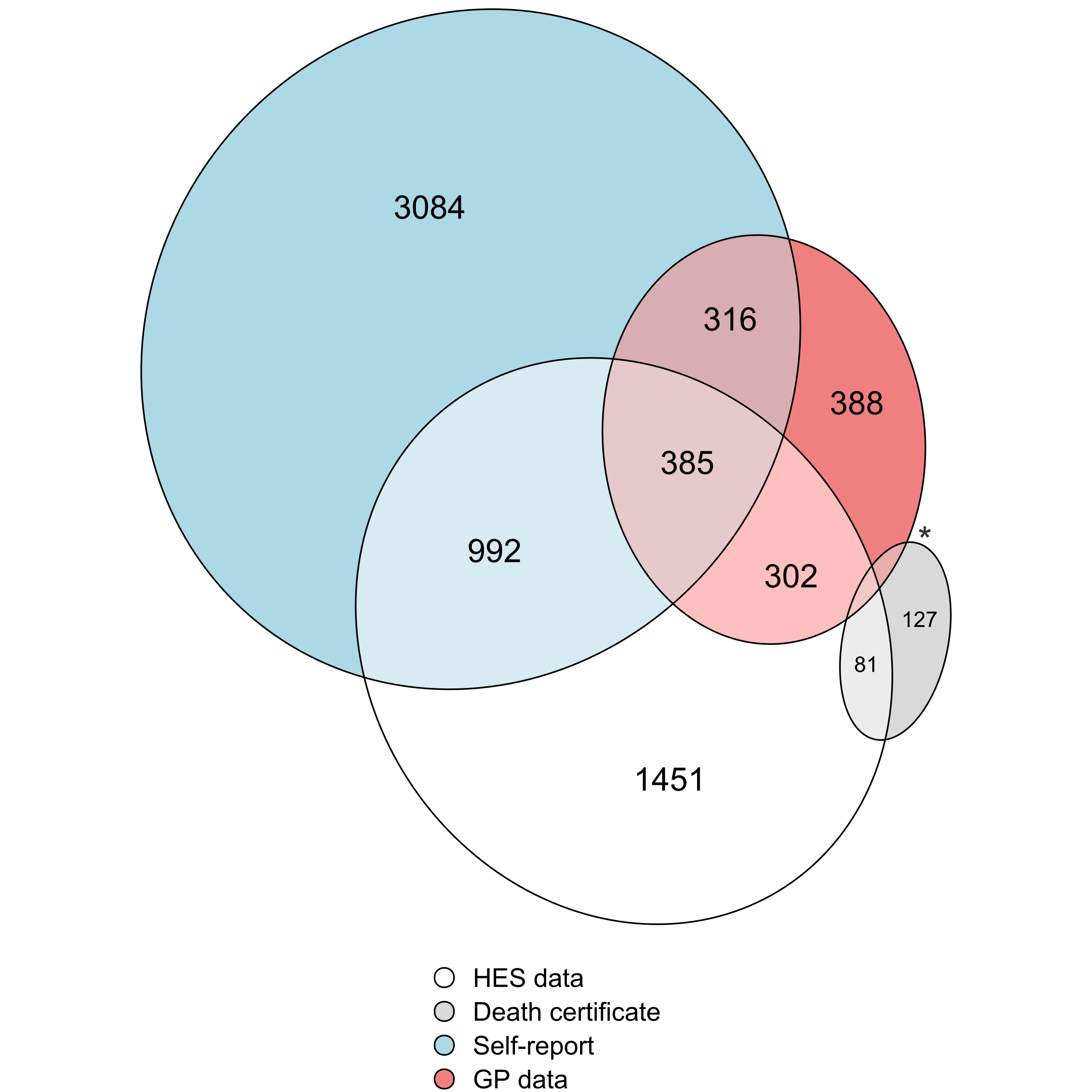


Web Figure 3 Venn diagram of the proportional overlap in VTE cases between different data sources restricted to the primary care cohort in UK Biobank including death certificate data. Sets with <20 participants have not been labelled. The asterisk indicates that this diagram is not accurate for the overlap of death certificate data – there are very small numbers of overlap with the self-report data that are not possible to display. See Web table 5 for exact numbers in these categories.


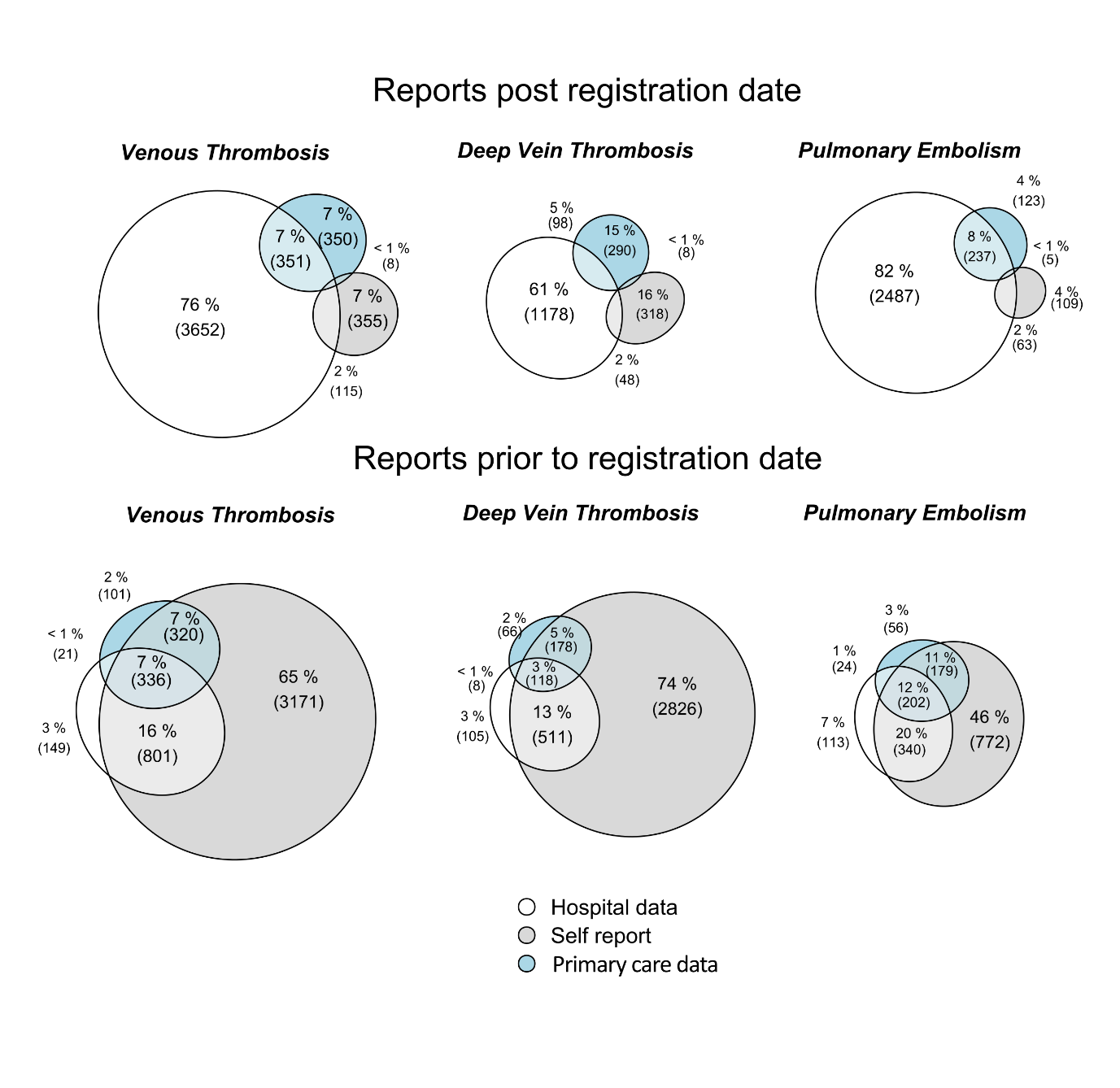


Web Figure 4 Venn diagram of the proportional overlap in VTE, DVT and PE cases in the primary care cohort from the difference reporting sources, split into events prior to registration and events post registration. Death Certificate data was not included, see Web Table 7 for incident events including death certificate. Self reported event post baseline were only available for a smaller proportion of UK Biobank who volunteered for follow up studies. See Web table 10 for exact numbers in these categories.


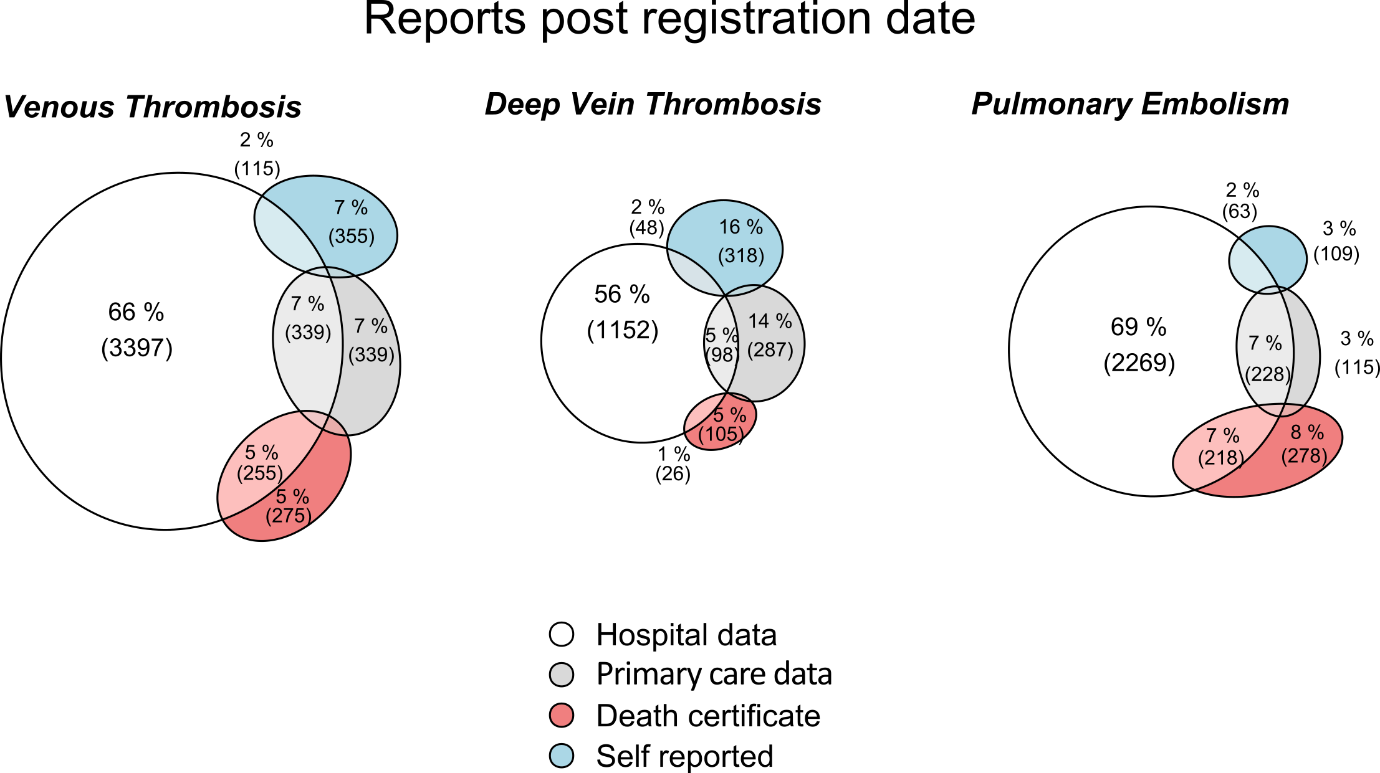


Web Figure 5 Venn diagram of the proportional overlap in VTE, DVT and PE cases in the primary care cohort from the difference reporting sources, including death certificate split into events prior to registration and events post registration. Categories with <1% of events in have not been labelled for clarity. Self-reported event post baseline were only available for a smaller proportion of UK Biobank who volunteered for follow up studies. See Web table 11 for exact numbers in these categories.


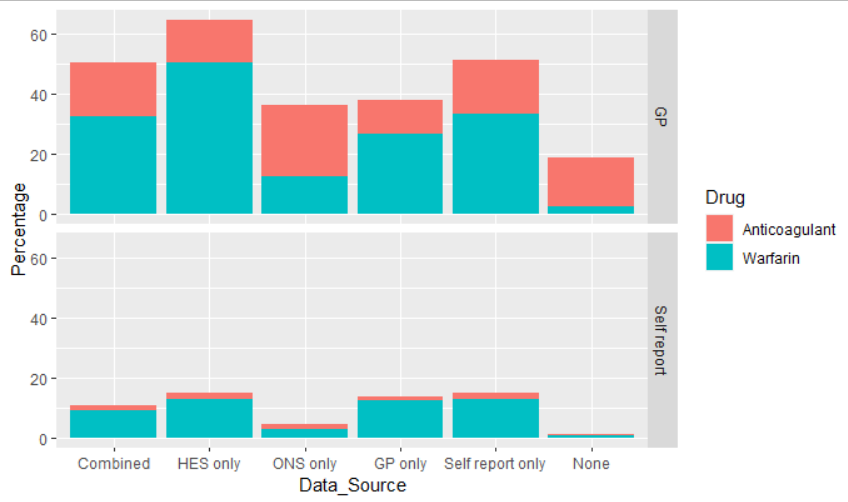


Web Figure 6 Difference in percentages of VTE cases reporting usage of general anticoagulants or warfarin split. The columns show the source of definition for the VTE case, while the two rows show whether the drug use was self-reported or in GP notes.

Web Table 1 Definitions of Venous thromboembolism (VTE), Pulmonary embolism (PE) and Deep venous thrombosis (DVT)

|  | HES & Death Registry data | | | Self-report fields | | Primary care fields | |
| --- | --- | --- | --- | --- | --- | --- | --- |
|  | ICD-9 codes | ICD-10 codes | Procedures OPCS4 | UKB 6152 | UKB 20002 | Read V3 Codes | Read V2 Codes |
|  |  |  |  |  |  |  |  |
| Venous thromboembolism | 415.1.X, 451.1.X, 452.X, 453.0, 453.4.X, 453.9.X, | I26.X, I80.1, I80.2, I81.X, I82.0 | L90.2 | 5,7 | 1068, 1093, 1094 | G8010, G8011, G8012, G8013, G8014, G8015, G8016, G8017, G8018, G8019, G801A, G81.X, G40.X, G400.X, G402.X, G40z.X | G40.X, G400.X, G401.X, G4010, G4011, G402.X, G40z.X, G801.X, G8010, G8011, G8012, G8013, G8014, G8015, G8016, G8017, G8018, G8019, G801A, G801B, G801C, G801D, G801E, G801F, G801G, G801H, G801J, G80y0, G80y1, G80y2, G80y3, G80y4, G80y5, G80y6, G80y7, G80y8 |
| Deep venous thrombosis | 451.X | I80.2 | L90.2 | 5 | 1094 | G8011, G8012, G8013, G8014, G8015, G8017, G8018, G8019, G801A | G801.X, G8011, G8012, G8013, G8014, G8015, G8017, G8018, G8019, G801A, G801B, G801C, G801D, G801E, G801F, G801G, G801H, G801J |
| Pulmonary embolism | 415.1.X | I26.X |  | 7 | 1093 | G40.X, G400.X, G402.X, G40z.X | G40.X, G400.X, G401.X, G4010, G4011, G402.X, G40z.X |

Web Table 2 List of names of medications marched with GP and self-report data, together with counts of each match found. The list of anticoagulants searched for were :Warfarin, Heparin, Indandione, Dalteparin, Coumadin, K-vitamin antagonist, Acenocoumarol and Aspirin

| Anti Coagulants matched to UK Biobank field 20003 | Number of Participants taking |
| --- | --- |
| warfarin | 5395 |
| aspirin 75mg tablet | 3552 |
| sodium warfarin | 422 |
| clexane 20mg/0.2ml prefilled syringe | 37 |
| heparin | 19 |
| enoxaparin | 17 |
| phenindione | 17 |
| fragmin 10,000iu/1ml injection | 14 |
| tinzaparin | 13 |
| aspirin+codeine | 11 |
| acenocoumarol | 7 |
| subcutaneous heparin | 4 |
| dalteparin | 3 |
| innohep 5000iu/0.5ml injection amp | 3 |
| aspirin+metoclopramide 325mg/5mg effervescent tablet | 2 |
| aspirin+codeine 300mg/8mg tablet | 1 |
| aspirin+methocarbamol 325mg/400mg tablet | 1 |
| dindevan 10mg tablet | 1 |

| Anti Coagulants matched to GP data | Number of Participants taking |
| --- | --- |
| Aspirin 75mg dispersible tablets | 27081 |
| Aspirin 75mg gastro-resistant tablets | 8076 |
| Warfarin 1mg tablets | 6674 |
| Warfarin 3mg tablets | 6610 |
| Aspirin 75mg tablets | 6146 |
| Warfarin 5mg tablets | 3513 |
| ASPIRIN disp tab 75mg | 1937 |
| Aspirin Dispersible tablets 75 mg | 1869 |
| Warfarin 500microgram tablets | 1862 |
| Aspirin 300mg dispersible tablets | 1355 |
| Aspirin Dispersible TABS 75MG | 828 |
| ASPIRIN DISPERSIBLE TABLETS 75MG | 704 |
| Aspirin 300mg tablets | 623 |
| ASPIRIN ec tab 75mg | 606 |
| Aspirin 300mg gastro-resistant tablets | 479 |
| WARFARIN SODIUM tabs 1mg | 475 |
| WARFARIN SODIUM tabs 3mg | 442 |
| ASPIRIN tabs 75mg | 385 |
| Warfarin Sodium Tablets 1 mg | 343 |
| Warfarin Sodium Tablets 3 mg | 319 |
| Aspirin TABS 75MG | 253 |
| Aspirin Tablets 75 mg | 232 |
| WARFARIN SODIUM tabs 5mg | 220 |
| Aspirin Ec TABS 75MG | 189 |
| Warfarin Sodium Tablets 5 mg | 186 |
| ASPIRIN EC TABLETS 75MG | 177 |
| Aspirin E/c tablets 75 mg | 172 |
| Dalteparin sodium 5,000units/0.2ml solution for injection pre-filled syringes | 172 |
| ASPIRIN TABLETS 75MG | 164 |
| Dalteparin sodium 18,000units/0.72ml solution for injection pre-filled syringes | 127 |
| Dalteparin sodium 15,000units/0.6ml solution for injection pre-filled syringes | 125 |
| Warfarin Sodium TABS 1MG | 115 |
| Warfarin Sodium TABS 3MG | 112 |
| Heparin sodium 50units/5ml patency solution ampoules | 85 |
| Dipyridamole 200mg modified-release / Aspirin 25mg capsules | 78 |
| Warfarin Sodium TABS 5MG | 78 |
| WARFARIN SODIUM TABLETS 1MG | 71 |
| Dalteparin sodium 12,500units/0.5ml solution for injection pre-filled syringes | 67 |
| Aspirin Dispersible tablets 300 mg | 61 |
| WARFARIN SODIUM TABLETS 3MG | 61 |
| ASPIRIN disp tab 300mg | 59 |
| ASPIRIN tabs 300mg | 52 |
| Dalteparin sodium 10,000units/0.4ml solution for injection pre-filled syringes | 52 |
| Aspirin 900mg / Metoclopramide 10mg oral powder sachets sugar free | 49 |
| Dalteparin sodium 5 000units/0.2ml solution for injection... | 45 |
| Warfarin Sodium Tablets 500 micrograms | 40 |
| WARFARIN SODIUM TABLETS 5MG | 40 |
| WARFARIN SODIUM tabs 500 micrograms | 38 |
| Dalteparin sodium 7,500units/0.3ml solution for injection pre-filled syringes | 37 |
| ASPIRIN ec tab 300mg | 35 |
| Warfarin 1mg tablets (Teva UK Ltd) | 31 |
| ASPIRIN dispersible tablet 75mg | 30 |
| heparin flush solution 10 units/ml | 30 |
| Aspirin Tablets 300 mg | 29 |
| ASPIRIN TAB 75mg | 29 |
| Heparin sodium 50units/5ml I.V. flush solution ampoules (Wockhardt UK Ltd) | 29 |
| WARFARIN TAB 1mg | 29 |
| Dalteparin sodium 12 500units/0.5ml solution for injectio... | 28 |
| Aspirin Dispersible Tablets 75 mg | 27 |
| ASPIRIN DISPERSIBLE TAB 75mg | 27 |
| Aspirin Dispersible Tablets 75 mg | 27 |
| WARFARIN WBP tablets 1mg [BOEH I HSP] | 27 |
| aspirin dispersible tablet 75mg | 26 |
| Aspirin Dispersible TABS 300MG | 26 |
| Dalteparin sodium 18 000units/0.72ml solution for injecti... | 24 |
| WARFARIN WBP tablets 3mg [BOEH I HSP] | 24 |
| Aspirin 75mg dispersible tablets (Teva UK Ltd) | 22 |
| ASPIRIN DISPERSIBLE TABLETS 300MG | 22 |
| Aspirin Dispersible TABLETS 75MG | 22 |
| WARFARIN TAB 3mg | 22 |
| co-codaprin (codeine and aspirin) tablets 8mg+400mg | 21 |
| Dalteparin sodium 2,500units/0.2ml solution for injection pre-filled syringes | 21 |
| Acenocoumarol 1mg tablets | 20 |
| Dalteparin sodium 15 000units/0.6ml solution for injectio... | 20 |
| Dalteparin sodium 2 500units/0.2ml solution for injection... | 20 |
| ASPIRIN DISPERSIBLE TAB 75 | 19 |
| WARFARIN WBP tablets 5mg [BOEH I HSP] | 19 |
| Aspirin E/C Tablets 75 mg | 18 |
| Aspirin dispersible tablet 75mg | 18 |
| Aspirin TABS 300MG | 18 |
| Warfarin 500microgram tablets (A A H Pharmaceuticals Ltd) | 18 |
| Warfarin 500microgram tablets (Actavis UK Ltd) | 18 |
| Aspirin 75mg dispersible tablets (Actavis UK Ltd) | 17 |
| Dalteparin Sodium Injection 18 000 units/0.72 ml pre-filled syringe | 17 |
| Warfarin 3mg tablets (Teva UK Ltd) | 16 |
| WARFARIN TAB 5mg | 16 |
| ASPIRIN TAB 75 | 15 |
| Aspirin 75mg gastro-resistant tablets (Almus Pharmaceuticals Ltd) | 14 |
| Aspirin 75mg gastro-resistant tablets (Sandoz Ltd) | 13 |
| ASPIRIN EC TABLETS 300MG | 13 |
| Aspirin Ec TABS 300MG | 13 |
| Dalteparin Sodium Injection 15 000 units/0.6 ml pre-filled syringe | 13 |
| Aspirin 75mg gastro-resistant tablets (Teva UK Ltd) | 12 |
| Dalteparin sodium 10 000units/1ml solution for injection ... | 12 |
| ASPIRIN 75mg dispersible tablets | 11 |
| ASPIRIN E/C TAB 300mg | 11 |
| Dalteparin Sodium Injection 2 500 units/0.2 ml pre-filled syringe | 11 |
| HEPARIN flush soln 10 i.u./ml | 11 |
| Aspirin E/c tablets 300 mg | 10 |
| Aspirin 75mg dispersible tablets (Almus Pharmaceuticals Ltd) | 10 |
| Aspirin E/C Tablets 75 mg | 10 |
| ASPIRIN TAB 300mg | 10 |
| ASPIRIN tablets 75mg | 10 |
| Dalteparin sodium 5,000units/0.2ml solution for injection... | 10 |
| DIPYRIDAMOLE + ASPIRIN mr cap 200mg + 25mg | 10 |
| Aspirin 75mg gastro-resistant tablets (Actavis UK Ltd) | 9 |
| ASPIRIN enteric coated tablets 75mg | 9 |
| Aspirin TABLETS 75MG | 9 |
| Dalteparin Sodium Injection 12 500 units/0.5 ml pre-filled syringe | 9 |
| Dalteparin sodium 10,000units/4ml solution for injection ampoules | 9 |
| HEPARIN flush solution 50 units/5ml [WOCKHARDT] | 9 |
| Aspirin 300mg effervescent tablets sugar free | 8 |
| Aspirin 500mg / Codeine 8mg dispersible tablets sugar free | 8 |
| ASPIRIN DISP TAB 75MG | 8 |
| ASPIRIN TABLETS 300MG | 8 |
| Warfarin 5mg tablets (Teva UK Ltd) | 8 |
| Warfarin Sodium Tablets 3 mg | 8 |
| WARFARIN TAB 1 | 8 |
| Aspirin 75mg gastro-resistant tablets (Wockhardt UK Ltd) | 7 |
| ASPIRIN E/C TAB 300 | 7 |
| ASPIRIN E/C TAB 75 | 7 |
| Aspirin Tablets 75 mg | 7 |
| Dalteparin Sodium Injection 5 000 units/0.2 ml pre-filled syringe | 7 |
| NU-SEALS ASPIRIN EC tablets 75mg [LILLY] | 7 |
| Warfarin 3mg tablets (Actavis UK Ltd) | 7 |
| Warfarin Sodium Tablets 1 mg | 7 |
| WARFARIN SODIUM tablets 3mg | 7 |
| Aspirin Ec TABLETS 75MG | 6 |
| aspirin enteric coated tablets 75mg | 6 |
| ASPIRIN TABS 75MG | 6 |
| dalteparin (form not specified) | 6 |
| DALTEPARIN inj soln 5000iu/0.2ml | 6 |
| Dalteparin sodium 7 500units/0.3ml solution for injection... | 6 |
| heparin sodium solution 10 units/ml | 6 |
| Warfarin 3mg tablets (Arrow Generics Ltd) | 6 |
| Warfarin 5mg tablets (Arrow Generics Ltd) | 6 |
| WARFARIN TAB 3 | 6 |
| Warfarin tablets 3mg | 6 |
| Aspirin 75mg gastro-resistant tablets (A A H Pharmaceuticals Ltd) | 5 |
| ASPIRIN DISPERSIBLE TAB 300mg | 5 |
| aspirin modified release tablet 100mg | 5 |
| Dalteparin sodium 10 000units/0.4ml solution for injectio... | 5 |
| Heparin Sodium I.V. Flush Solution for injection 10 units/ml 5 ml ampoule | 5 |
| Warfarin 1mg tablets (Almus Pharmaceuticals Ltd) | 5 |
| Warfarin Sodium TABLETS 1MG | 5 |
| WARFARIN SODIUM tablets 1mg | 5 |
| WARFARIN SODIUM TABS 1MG | 5 |
| Warfarin Sodium TABS 500MICROGRAMS | 5 |
| Warfarin tablets 1mg | 5 |
| Aspirin 300mg dispersible tablets (Almus Pharmaceuticals Ltd) | 4 |
| Aspirin 75 mg soluble tablets | 4 |
| Aspirin 75mg dispersible tablets (A A H Pharmaceuticals Ltd) | 4 |
| ASPIRIN 75MG TABLET | 4 |
| ASPIRIN DISP TAB 300MG | 4 |
| ASPIRIN E/C TAB 75mg | 4 |
| ASPIRIN SOLUBLE 75mg | 4 |
| ASPIRIN tabs, dispersible 75 mg | 4 |
| Dalteparin Sodium Injection 25000 units/ml 0.72 ml (18000 units) syringe | 4 |
| Dalteparin sodium 10,000units/4ml solution for injection ... | 4 |
| Dalteparin sodium 15,000units/0.6ml solution for injectio... | 4 |
| Warfarin 3mg tablets (Almus Pharmaceuticals Ltd) | 4 |
| Warfarin 5mg tablets (Actavis UK Ltd) | 4 |
| Warfarin 5mg tablets (Almus Pharmaceuticals Ltd) | 4 |
| Warfarin Sodium TABLETS 3MG | 4 |
| Warfarin Sodium Tablets 5 mg | 4 |
| Warfarin Sodium TABLETS 5MG | 4 |
| WARFARIN SODIUM TABS 3MG | 4 |
| WARFARIN SODIUM TABS 5MG | 4 |
| ASPIRIN | 3 |
| Aspirin E/C Tablets 300 mg | 3 |
| Aspirin 300mg dispersible tablets (Aspar Pharmaceuticals Ltd) | 3 |
| ASPIRIN 300mg e/c tablets | 3 |
| Aspirin 300mg gastro-resistant tablets (A A H Pharmaceuticals Ltd) | 3 |
| Aspirin 300mg modified-release tablets | 3 |
| Aspirin 500mg / Papaveretum 7.71mg dispersible tablets sugar free | 3 |
| ASPIRIN 75mg disp tabs | 3 |
| Aspirin 75mg dispersible tablets (Kent Pharmaceuticals Ltd) | 3 |
| ASPIRIN 75mg e/c tablets | 3 |
| ASPIRIN 75mg soluble tablets | 3 |
| Aspirin 75mg tablets (A A H Pharmaceuticals Ltd) | 3 |
| Aspirin Disp 75mg Tabs | 3 |
| ASPIRIN EC TAB 75MG | 3 |
| Aspirin enteric coated tablets 75mg | 3 |
| DALTEPARIN inj soln 2500iu/0.2ml | 3 |
| Dalteparin Sodium Injection 10 000 units/0.4 ml pre-filled syringe | 3 |
| Dalteparin Sodium Injection 12500 units/ml 0.2 ml (2500 units) syringe | 3 |
| Dalteparin Sodium Injection 7 500 units/0.3 ml pre-filled syringe | 3 |
| Dalteparin sodium 10,000units/1ml solution for injection ... | 3 |
| Dalteparin sodium 10,000units/1ml solution for injection ampoules | 3 |
| Dalteparin sodium 12,500units/0.5ml solution for injectio... | 3 |
| Dalteparin sodium 18,000units/0.72ml solution for injecti... | 3 |
| Heparin 10 iu/ml Flush solution | 3 |
| Heparin sodium 200units/2ml patency solution ampoules | 3 |
| Heparin sodium 5,000units/5ml solution for injection ampoules | 3 |
| Heparin sodium 50units/5ml I.V. flush solution ampoules (LEO Pharma) | 3 |
| HEPARIN SODIUM flush soln 50 i.u./5ml | 3 |
| Nu-seals aspirin ec 75mg Gastro-resistant tablet (Eli Lilly and Company Ltd) | 3 |
| NU-SEALS ASPIRIN EC tab 75mg | 3 |
| NUSEALS ASPIRIN EC TABLETS 300MG | 3 |
| Nuseals Aspirin Gastro Resistant TABS 300MG | 3 |
| Nuseals Aspirin Gastro Resistant TABS 75MG | 3 |
| Warfarin sodium tablets 1mg | 3 |
| WARFARIN SODIUM tablets 5mg | 3 |
| WARFARIN SODIUM tabs 1 mg | 3 |
| ACENOCOUMAROL tabs 1mg | 2 |
| Aspirin-P42 300mg tab EC | 2 |
| Aspirin-P42 75mg Tab Soluble | 2 |
| Aspirin 150mg suppositories | 2 |
| Aspirin 300mg dispersible tablets (A A H Pharmaceuticals Ltd) | 2 |
| Aspirin 300mg dispersible tablets (Actavis UK Ltd) | 2 |
| Aspirin 300mg tablets (Actavis UK Ltd) | 2 |
| ASPIRIN 75MG DISP TABLETS | 2 |
| Aspirin 75mg gastro-resistant tablets (Kent Pharmaceuticals Ltd) | 2 |
| Aspirin And Metoclopramide Oral Powder Sachets Sugar Free 900 mg + 10 mg | 2 |
| Aspirin Dispersible Low Dose Tablets 75 mg | 2 |
| Aspirin Dispersible Tablet 75mg | 2 |
| Aspirin Dispersible Tablets 300 mg | 2 |
| Aspirin E/C Tablets 300 mg | 2 |
| ASPIRIN EC 75MG CAPSULE | 2 |
| ASPIRIN EC 75MG TAB | 2 |
| ASPIRIN ec tabs 75mg | 2 |
| ASPIRIN NON TARIFF EC TABLETS 75MG | 2 |
| ASPIRIN SOLUBLE TAB 300 | 2 |
| ASPIRIN SOLUBLE TAB 300mg | 2 |
| ASPIRIN TAB 300 | 2 |
| dalteparin injection solution 10000 units/1ml | 2 |
| DALTEPARIN SODIUM SYRINGE 0.4ML INJ 10000UNITS | 2 |
| Dalteparin Sodium Syringe 0.72ml INJ 18000UNITS | 2 |
| HEPARIN flush soln 10 units/ml | 2 |
| heparin flush solution 100 units/ml | 2 |
| Heparin sodium 5,000units/5ml solution for injection vials | 2 |
| Heparin sodium 50units/5ml I.V. flush solution ampoules (... | 2 |
| Heparin Sodium I.V. Flush Solution for injection 100 units/ml 2 ml ampoule | 2 |
| metoclopramide with aspirin effervescent tablet 5mg + 325mg | 2 |
| metoclopramide with aspirin effervescent tablet 5mg + 450mg | 2 |
| NU-SEALS ASPIRIN EC tab 300mg | 2 |
| NU-SEALS ASPIRIN EC tablets 300mg [LILLY] | 2 |
| NUSEALS ASPIRIN EC TABLETS 75MG | 2 |
| SOL ASPIRIN | 2 |
| WARFARIN 1mg tablets | 2 |
| Warfarin 1mg tablets (Actavis UK Ltd) | 2 |
| Warfarin 3mg tablets (IVAX Pharmaceuticals UK Ltd) | 2 |
| Warfarin 3mg/5ml oral solution | 2 |
| WARFARIN SOD TAB 1MG | 2 |
| WARFARIN SOD TAB 3MG | 2 |
| WARFARIN SOD TAB 5MG | 2 |
| WARFARIN SODIUM | 2 |
| WARFARIN SODIUM 1mg tablets | 2 |
| WARFARIN SODIUM 3mg tablets | 2 |
| WARFARIN SODIUM tabs 5 mg | 2 |
| Warfarin tablets 5mg | 2 |
| Acenocoumarol Tablets 1 mg | 1 |
| Aspirin-P42 300mg tablet | 1 |
| Aspirin-P42 75mg Tablet | 1 |
| ASPIRIN-P42 75MG TABLET | 1 |
| Aspirin Dispersible Tablets 300 mg | 1 |
| Aspirin M/R tablets 100 mg | 1 |
| ASPIRIN & PAPAVERUTUM TAB 10mg | 1 |
| ASPIRIN (Generic Manuf) (form not specified) | 1 |
| ASPIRIN + CODEINE tabs 400mg + 8mg | 1 |
| ASPIRIN 300 MG E/C TAB | 1 |
| Aspirin 300 mg enteric coated tablets | 1 |
| ASPIRIN 300mg disp tablets | 1 |
| Aspirin 300mg gastro-resistant tablets (Mylan Ltd) | 1 |
| Aspirin 300mg gastro-resistant tablets (Sandoz Ltd) | 1 |
| Aspirin 300mg gastro-resistant tablets (Teva UK Ltd) | 1 |
| ASPIRIN 300mg soluble tablets | 1 |
| Aspirin 300mg suppositories | 1 |
| Aspirin 300mg tablets (Aspar Pharmaceuticals Ltd) | 1 |
| Aspirin 300mg tablets (Sigma Pharmaceuticals Plc) | 1 |
| Aspirin 325mg / Caffeine 15mg tablets | 1 |
| Aspirin 400mg with Codeine 8mg tablets | 1 |
| Aspirin 500mg effervescent tablets sugar free | 1 |
| Aspirin 75 mg dispersible tablets | 1 |
| Aspirin 75 mg enteric coated tablets | 1 |
| Aspirin 75 mg tablets | 1 |
| ASPIRIN 75mg | 1 |
| ASPIRIN 75MG | 1 |
| ASPIRIN 75MG DISP TABS | 1 |
| Aspirin 75mg dispersible tablets (Aspar Pharmaceuticals Ltd) | 1 |
| ASPIRIN 75MG TAB | 1 |
| Aspirin 900mg / Metoclopramide 10mg oral powder sachets suga | 1 |
| ASPIRIN Disp | 1 |
| ASPIRIN DISP 75MG | 1 |
| ASPIRIN disp tab 75 MG | 1 |
| Aspirin Dispersible Low Dose Tablets 75 mg | 1 |
| ASPIRIN DISPERSIBLE TAB 300 | 1 |
| Aspirin Dispersible Table | 1 |
| Aspirin dispersible tablet 300mg | 1 |
| aspirin dispersible tablet 75 mg | 1 |
| ASPIRIN dispersible tablet 75mg[tab blister pack] | 1 |
| Aspirin Dispersible tablets 75mg | 1 |
| Aspirin Dispersible Tablets 75mg | 1 |
| ASPIRIN E/C TABLETS 75-P42 0 | 1 |
| ASPIRIN EC 300 MGM TABS | 1 |
| ASPIRIN EC 300MG | 1 |
| ASPIRIN EC 75 MGMS TABLET | 1 |
| ASPIRIN EC 75MG | 1 |
| ASPIRIN EC TAB 300MG | 1 |
| aspirin ec tablets 300mg | 1 |
| aspirin ec tablets 75mg | 1 |
| ASPIRIN eff tab 300mg | 1 |
| ASPIRIN EFFERVESCENT tabs 100 MG | 1 |
| Aspirin enteric coated tablets 300mg | 1 |
| ASPIRIN ENTERIC COATED TABLETS 75MG | 1 |
| ASPIRIN enteric coated tablets 75mg [GALEN] | 1 |
| aspirin modified release tablet 324mg | 1 |
| ASPIRIN NUSEAL 75MG | 1 |
| ASPIRIN NUSEAL 75MG TABLET | 1 |
| ASPIRIN NUSEALS 300MG | 1 |
| ASPIRIN NUSEALS TAB 300 | 1 |
| ASPIRIN PAED 75MG TABLETS | 1 |
| ASPIRIN S/R 600mg | 1 |
| ASPIRIN SOLUBLE 75MG TABS | 1 |
| ASPIRIN TAB 150mg | 1 |
| ASPIRIN TABLETS DISPERSIBLE 300MG-P42 0 | 1 |
| ASPIRIN TABLETS 300 MG | 1 |
| Aspirin tablets 300mg | 1 |
| ASPIRIN tablets 300mg [CO-OPERATI] | 1 |
| aspirin tablets 75 mg | 1 |
| ASPIRIN TABLETS 75 MG | 1 |
| Aspirin tablets 75mg | 1 |
| Aspirin Tablets 75mg | 1 |
| Aspirin Tablets disp. 75 mg | 1 |
| ASPIRIN TABS 300MG | 1 |
| ASPIRIN TABS 75MG EC | 1 |
| ASPIRIN tabs dispersible 75 mg | 1 |
| ASPIRIN tabs e/c 75 mg | 1 |
| ASPIRIN tabs, e/c 75 mg | 1 |
| ASPIRIN TABS, E/C 75 MG | 1 |
| Aspirin with codeine tablets 400mg + 8mg | 1 |
| asprin 75mg | 1 |
| ASPRIN TABS DISPERSIBLE-P42 75mg | 1 |
| CO-CODAPRIN (codeine & aspirin) sol tab 8mg+500mg | 1 |
| DALTEPARIN inj soln 10000 iu/0.4ml | 1 |
| DALTEPARIN inj soln 10000 iu/1ml | 1 |
| DALTEPARIN inj soln 12500 iu/0.5ml | 1 |
| DALTEPARIN inj soln 18000 iu/0.72ml | 1 |
| DALTEPARIN inj soln 7500 iu/0.3ml | 1 |
| dalteparin injection solution 25000 units/ml | 1 |
| Dalteparin Sodium Injection 10 000 units/1 ml ampoule | 1 |
| Dalteparin Sodium Injection 25000 units/ml 0.2 ml (5000 units) syringe | 1 |
| Dalteparin Sodium Injection 25000 units/ml 0.4 ml (10000 units) syringe | 1 |
| Dalteparin Sodium Injection 25000 units/ml 0.6 ml (15000 units) syringe | 1 |
| Dalteparin Sodium Injection 5,000 units/0.2 ml pre-filled | 1 |
| Dalteparin sodium 10,000units/0.4ml solution for injectio... | 1 |
| Dalteparin sodium 10,000units/1ml solution for injection pre-filled syringes | 1 |
| Dalteparin sodium 2,500units/0.2ml solution for injection... | 1 |
| Dalteparin sodium 7,500units/0.3ml solution for injection... | 1 |
| DALTEPARIN SODIUM SYRINGE 0.2ML INJ 2500UNITS | 1 |
| Dalteparin Sodium Syringe 0.3ml INJ 7500UNITS | 1 |
| Dalteparin Sodium Syringe 0.4ml INJ 10000UNITS | 1 |
| DALTEPARIN SODIUM SYRINGE 0.5ML INJ 12500UNITS | 1 |
| Dalteparin Sodium Syringe 0.6ml INJ 15000UNITS | 1 |
| DALTEPARIN SODIUM SYRINGE 0.72ML INJ 18000UNITS | 1 |
| DALTEPARIN sterile soln 10000 iu/ml | 1 |
| DALTEPARIN sterile soln 2500 iu/ml | 1 |
| DALTEPARIN sterile soln 2500iu/0.2ml | 1 |
| DALTEPARIN sterile soln 5000iu/0.2ml | 1 |
| Dipyridamole & Aspirin 200 mg + 25 mg modified release capsu | 1 |
| heparin (form not specified) | 1 |
| Heparin Sodium Patency Solution 100 units/ml 2 ml ampoule | 1 |
| Heparin sodium 1,000units/1ml solution for injection ampo... | 1 |
| Heparin sodium 1,000units/1ml solution for injection ampoules | 1 |
| Heparin sodium 200units/2ml I.V. flush solution ampoules (Wockhardt UK Ltd) | 1 |
| Heparin sodium 5,000units/1ml solution for injection ampoules | 1 |
| Heparin sodium 5,000units/5ml solution for injection ampoules (LEO Pharma) | 1 |
| heparin sodium subcutaneous injection 25000 units/ml | 1 |
| HEPARIN solution 100 units/ml [LEO] | 1 |
| MEPROBAMATE + ETHOHEPTAZINE CITRATE & ASPIRIN tabs | 1 |
| meprobamate with ethoheptazine citrate and aspirin tablets | 1 |
| METOCLOPRAMIDE HYD. 5mg/325mg ASPIRIN TAB | 1 |
| METOCLOPRAMIDE HYD. 5mg/450mg ASPIRIN TAB | 1 |
| Nu-Seals Aspirin E/C Tablets 300 mg | 1 |
| Nu-Seals Aspirin E/c tablets 75 mg | 1 |
| Nu-Seals Aspirin E/C Tablets 75 mg | 1 |
| Nu-seals aspirin ec 300mg Gastro-resistant tablet (Eli Lilly and Company Ltd) | 1 |
| NU-SEALS ASPIRIN EC tabs 75mg | 1 |
| NU-SEALS ASPIRIN TAB 300mg | 1 |
| NU-SEALS ASPIRIN tablets 600mg [LILLY] | 1 |
| nuseal aspirin 300mg tab | 1 |
| SOLUBLE ASPIRIN | 1 |
| Soluble Aspirin Paediatric Tablets 75 mg | 1 |
| WARFARIN | 1 |
| WARFARIN 1MG | 1 |
| Warfarin 1mg Tablet (WB Pharmaceuticals Ltd) | 1 |
| Warfarin 1mg tablets (A A H Pharmaceuticals Ltd) | 1 |
| Warfarin 1mg tablets (Arrow Generics Ltd) | 1 |
| Warfarin 1mg tablets (IVAX Pharmaceuticals UK Ltd) | 1 |
| Warfarin 1mg tablets (Sandoz Ltd) | 1 |
| Warfarin 1mg/ml oral suspension sugar free | 1 |
| Warfarin 3mg Tablet (WB Pharmaceuticals Ltd) | 1 |
| WARFARIN 3mg tablets | 1 |
| WARFARIN 3MG TABLETS | 1 |
| Warfarin 3mg tablets (A A H Pharmaceuticals Ltd) | 1 |
| Warfarin 3mg tablets (Sandoz Ltd) | 1 |
| WARFARIN 5MG | 1 |
| WARFARIN 5mg tablets | 1 |
| Warfarin 5mg tablets (A A H Pharmaceuticals Ltd) | 1 |
| Warfarin 5mg tablets (IVAX Pharmaceuticals UK Ltd) | 1 |
| Warfarin 5mg tablets (Zentiva) | 1 |
| Warfarin 5mg/5ml oral solution | 1 |
| Warfarin Sodium Oral solution 1 mg/5 ml | 1 |
| Warfarin Sodium Oral solution 3 mg/5 ml | 1 |
| Warfarin sodium 1 mg tablets | 1 |
| WARFARIN SODIUM 1MG | 1 |
| WARFARIN SODIUM 3MG TAB | 1 |
| WARFARIN SODIUM 5mg tablets | 1 |
| WARFARIN SODIUM oral liq 3mg/5ml | 1 |
| warfarin sodium oral suspension 5mg/5ml | 1 |
| warfarin sodium oral suspension sugar-free 1mg/ml | 1 |
| warfarin sodium tablets 1 mg | 1 |
| warfarin sodium tablets 1mg | 1 |
| Warfarin Sodium Tablets 1mg | 1 |
| warfarin sodium tablets 3mg | 1 |
| Warfarin sodium tablets 3mg | 1 |
| warfarin sodium tablets 5 mg | 1 |
| warfarin sodium tablets 500 micrograms | 1 |
| WARFARIN SODIUM TABLETS 500MICROGRAMS | 1 |
| Warfarin sodium tablets 5mg | 1 |
| Warfarin Sodium Tablets 5mg | 1 |
| WARFARIN SODIUM Tabs | 1 |
| WARFARIN SODIUM tabs 3 mg | 1 |
| WARFARIN TAB 5 | 1 |
| Warfarin Tablets 1 mg | 1 |
| WARFARIN tablets 1mg [AMCO] | 1 |
| Warfarin Tablets 3 mg | 1 |
| WARFARIN tabs 1mg [IVAX] | 1 |
| WARFARIN tabs 3mg [IVAX] | 1 |
| WARFARIN tabs 3mg [WB] | 1 |
| WARFARIN tabs 500 micrograms [HILLCROSS] | 1 |
| WARFARIN WBP 1mg tabs | 1 |
| WARFARIN WBP 5mg tabs | 1 |
| WARFARIN WBP TABLETS 1 MG | 1 |
| WARFARIN WBP TABLETS 3 MG | 1 |
|  |  |

| Warfarin matched to UK Biobank field 20003 | Number of Participants taking |
| --- | --- |
| warfarin | 5395 |

| Warfarin matched to GP data | Number of Participants taking |
| --- | --- |
| Warfarin 1mg tablets | 6674 |
| Warfarin 3mg tablets | 6610 |
| Warfarin 5mg tablets | 3513 |
| Warfarin 500microgram tablets | 1862 |
| WARFARIN SODIUM tabs 1mg | 475 |
| WARFARIN SODIUM tabs 3mg | 442 |
| Warfarin Sodium Tablets 1 mg | 343 |
| Warfarin Sodium Tablets 3 mg | 319 |
| WARFARIN SODIUM tabs 5mg | 220 |
| Warfarin Sodium Tablets 5 mg | 186 |
| Warfarin Sodium TABS 1MG | 115 |
| Warfarin Sodium TABS 3MG | 112 |
| Warfarin Sodium TABS 5MG | 78 |
| WARFARIN SODIUM TABLETS 1MG | 71 |
| WARFARIN SODIUM TABLETS 3MG | 61 |
| Warfarin Sodium Tablets 500 micrograms | 40 |
| WARFARIN SODIUM TABLETS 5MG | 40 |
| WARFARIN SODIUM tabs 500 micrograms | 38 |
| Warfarin 1mg tablets (Teva UK Ltd) | 31 |
| WARFARIN TAB 1mg | 29 |
| WARFARIN WBP tablets 1mg [BOEH I HSP] | 27 |
| WARFARIN WBP tablets 3mg [BOEH I HSP] | 24 |
| WARFARIN TAB 3mg | 22 |
| WARFARIN WBP tablets 5mg [BOEH I HSP] | 19 |
| Warfarin 500microgram tablets (A A H Pharmaceuticals Ltd) | 18 |
| Warfarin 500microgram tablets (Actavis UK Ltd) | 18 |
| Warfarin 3mg tablets (Teva UK Ltd) | 16 |
| WARFARIN TAB 5mg | 16 |
| Warfarin 5mg tablets (Teva UK Ltd) | 8 |
| Warfarin Sodium Tablets 3 mg | 8 |
| WARFARIN TAB 1 | 8 |
| Warfarin 3mg tablets (Actavis UK Ltd) | 7 |
| Warfarin Sodium Tablets 1 mg | 7 |
| WARFARIN SODIUM tablets 3mg | 7 |
| Warfarin 3mg tablets (Arrow Generics Ltd) | 6 |
| Warfarin 5mg tablets (Arrow Generics Ltd) | 6 |
| WARFARIN TAB 3 | 6 |
| Warfarin tablets 3mg | 6 |
| Warfarin 1mg tablets (Almus Pharmaceuticals Ltd) | 5 |
| Warfarin Sodium TABLETS 1MG | 5 |
| WARFARIN SODIUM tablets 1mg | 5 |
| WARFARIN SODIUM TABS 1MG | 5 |
| Warfarin Sodium TABS 500MICROGRAMS | 5 |
| Warfarin tablets 1mg | 5 |
| Warfarin 3mg tablets (Almus Pharmaceuticals Ltd) | 4 |
| Warfarin 5mg tablets (Actavis UK Ltd) | 4 |
| Warfarin 5mg tablets (Almus Pharmaceuticals Ltd) | 4 |
| Warfarin Sodium TABLETS 3MG | 4 |
| Warfarin Sodium Tablets 5 mg | 4 |
| Warfarin Sodium TABLETS 5MG | 4 |
| WARFARIN SODIUM TABS 3MG | 4 |
| WARFARIN SODIUM TABS 5MG | 4 |
| Warfarin sodium tablets 1mg | 3 |
| WARFARIN SODIUM tablets 5mg | 3 |
| WARFARIN SODIUM tabs 1 mg | 3 |
| WARFARIN 1mg tablets | 2 |
| Warfarin 1mg tablets (Actavis UK Ltd) | 2 |
| Warfarin 3mg tablets (IVAX Pharmaceuticals UK Ltd) | 2 |
| Warfarin 3mg/5ml oral solution | 2 |
| WARFARIN SOD TAB 1MG | 2 |
| WARFARIN SOD TAB 3MG | 2 |
| WARFARIN SOD TAB 5MG | 2 |
| WARFARIN SODIUM | 2 |
| WARFARIN SODIUM 1mg tablets | 2 |
| WARFARIN SODIUM 3mg tablets | 2 |
| WARFARIN SODIUM tabs 5 mg | 2 |
| Warfarin tablets 5mg | 2 |
| WARFARIN | 1 |
| WARFARIN 1MG | 1 |
| Warfarin 1mg Tablet (WB Pharmaceuticals Ltd) | 1 |
| Warfarin 1mg tablets (A A H Pharmaceuticals Ltd) | 1 |
| Warfarin 1mg tablets (Arrow Generics Ltd) | 1 |
| Warfarin 1mg tablets (IVAX Pharmaceuticals UK Ltd) | 1 |
| Warfarin 1mg tablets (Sandoz Ltd) | 1 |
| Warfarin 1mg/ml oral suspension sugar free | 1 |
| Warfarin 3mg Tablet (WB Pharmaceuticals Ltd) | 1 |
| WARFARIN 3mg tablets | 1 |
| WARFARIN 3MG TABLETS | 1 |
| Warfarin 3mg tablets (A A H Pharmaceuticals Ltd) | 1 |
| Warfarin 3mg tablets (Sandoz Ltd) | 1 |
| WARFARIN 5MG | 1 |
| WARFARIN 5mg tablets | 1 |
| Warfarin 5mg tablets (A A H Pharmaceuticals Ltd) | 1 |
| Warfarin 5mg tablets (IVAX Pharmaceuticals UK Ltd) | 1 |
| Warfarin 5mg tablets (Zentiva) | 1 |
| Warfarin 5mg/5ml oral solution | 1 |
| Warfarin Sodium Oral solution 1 mg/5 ml | 1 |
| Warfarin Sodium Oral solution 3 mg/5 ml | 1 |
| Warfarin sodium 1 mg tablets | 1 |
| WARFARIN SODIUM 1MG | 1 |
| WARFARIN SODIUM 3MG TAB | 1 |
| WARFARIN SODIUM 5mg tablets | 1 |
| WARFARIN SODIUM oral liq 3mg/5ml | 1 |
| warfarin sodium oral suspension 5mg/5ml | 1 |
| warfarin sodium oral suspension sugar-free 1mg/ml | 1 |
| warfarin sodium tablets 1 mg | 1 |
| warfarin sodium tablets 1mg | 1 |
| Warfarin Sodium Tablets 1mg | 1 |
| warfarin sodium tablets 3mg | 1 |
| Warfarin sodium tablets 3mg | 1 |
| warfarin sodium tablets 5 mg | 1 |
| warfarin sodium tablets 500 micrograms | 1 |
| WARFARIN SODIUM TABLETS 500MICROGRAMS | 1 |
| Warfarin sodium tablets 5mg | 1 |
| Warfarin Sodium Tablets 5mg | 1 |
| WARFARIN SODIUM Tabs | 1 |
| WARFARIN SODIUM tabs 3 mg | 1 |
| WARFARIN TAB 5 | 1 |
| Warfarin Tablets 1 mg | 1 |
| WARFARIN tablets 1mg [AMCO] | 1 |
| Warfarin Tablets 3 mg | 1 |
| WARFARIN tabs 1mg [IVAX] | 1 |
| WARFARIN tabs 3mg [IVAX] | 1 |
| WARFARIN tabs 3mg [WB] | 1 |
| WARFARIN tabs 500 micrograms [HILLCROSS] | 1 |
| WARFARIN WBP 1mg tabs | 1 |
| WARFARIN WBP 5mg tabs | 1 |
| WARFARIN WBP TABLETS 1 MG | 1 |
| WARFARIN WBP TABLETS 3 MG | 1 |

Web Table 3 Comparison of UK Biobank demographics with general UK population

|  | UK Biobank All  (n =502520) | UK Biobank | UK Populace | Ref for UK Populace figures |
| --- | --- | --- | --- | --- |
|  |  | Primary Care cohort  (n= 177358) |  |  |
| Gender (% Female) | 54.4 | 54.5 | 50.8 | 2011 UK Census, Table QS104EW |
| Median Age (years) | 58.3 | 58.4 | 40 | Humby P. Overview of the UK population: February 2016. Office for National Statistics. Accessed 10/11/2022, 2022. |
| Ethnicity (% White) | 94.6 | 95.7 | 86 | 2011 UK Census, Table KS201EW |
| Unemployment (%) | 43.1 | 44.1 | 34.7 | 2011 UK Census, Table QS601EW |
|  |  |  |  | (Combining unemployed and economically inactive) |
| Higher Education (%) | 60.2 | 59.8 | 32 | 2011UK Census, Table QS501EW |
|  |  |  |  | (Combining Level 4 and Other) |
| Mean BMI (female) | 27.1 | 27.2 | 28 | Health Survey for England 2011, data for women aged 45-54 |
|  |  |  |  | doi:10.5255/UKDA-SN-7260-1 |
| Mean BMI (male) | 27.8 | 27.9 | 28.6 | Health Survey for England 2011, data for men aged 45-54 |
|  |  |  |  | doi:10.5255/UKDA-SN-7260-1 |
| Current smokers (Women) (%) | 8.9 | 8.7 | 22 | Health Survey for England 2011, data for women aged 45-54 |
|  |  |  |  | doi:10.5255/UKDA-SN-7260-1 |
| Current Smokers (men) ( %) | 12.5 | 12.1 | 24 | Health Survey for England 2011, data for men aged 45-54 |
|  |  |  |  | doi:10.5255/UKDA-SN-7260-1 |

Web Table 4 Count of cases in UK Biobank broken down by source(s) of report

| All data  (n =502520) | All Venous Thromboembolism | % | Deep Vein Thrombosis only | % | Pulmonary Embolism only | % |
| --- | --- | --- | --- | --- | --- | --- |
| Hospital Episode Statistics only | 5118 | 26% | 2061 | 16% | 3521 | 42% |
| Death Certificate only | 388 | 2% | 199 | 2% | 407 | 5% |
| Self Report only | 9843 | 50% | 8695 | 66% | 2588 | 31% |
| Hospital Episode Statistics & Death Certificate | 291 | 1% | 40 | 0% | 248 | 3% |
| Hospital Episode Statistics & Self Report | 3822 | 20% | 2073 | 16% | 1658 | 20% |
| Death Certificate & Self Report | 21 | 0% | 9 | 0% | 11 | 0% |
| Hospital Episode Statistics & Death Certificate & Self Report | 41 | 0% | 7 | 0% | 18 | 0% |
|  |  |  |  |  |  |  |
| Appearing only in a single source | 15349 | 79% | 10955 | 84% | 6516 | 77% |
|  |  |  |  |  |  |  |
| Total events (Percentage of total VTEs) | 19524 | 100% | 13084 | 67% | 8451 | 43% |

Web Table 5 Count of cases in the Primary Care cohort broken down by source(s) of report

| Primary Care subset  (n= 177358) | All Venous Thromboembolism | % | Deep Vein Thrombosis only | % | Pulmonary Embolism only | % |
| --- | --- | --- | --- | --- | --- | --- |
| Hospital Episode Statistics only | 1451 | 20% | 559 | 12% | 1017 | 32% |
| Death Certificate only | 127 | 2% | 54 | 1% | 138 | 4% |
| Self Report only | 3084 | 43% | 2836 | 60% | 741 | 23% |
| Hospital Episode Statistics & Death Certificate | 81 | 1% | 10 | 0% | 72 | 2% |
| Hospital Episode Statistics & Self Report | 992 | 14% | 593 | 12% | 388 | 12% |
| Death Certificate & Self Report | 7 | 0% | 4 | 0% | 5 | 0% |
| Hospital Episode Statistics & Death Certificate & Self Report | 16 | 0% | 2 | 0% | 7 | 0% |
| Primary Care Data only | 388 | 5% | 290 | 6% | 159 | 5% |
| Hospital Episode Statistics & Primary Care Data | 302 | 4% | 83 | 2% | 222 | 7% |
| Death Certificate & Primary Care Data | 9 | 0% | 3 | 0% | 7 | 0% |
| Self Report & Primary Care Data | 316 | 4% | 190 | 4% | 167 | 5% |
| Hospital Episode Statistics & Death Certificate & Primary Care Data | 9 | 0% | 0 | 0% | 7 | 0% |
| Hospital Episode Statistics & Self Report & Primary Care Data | 385 | 5% | 131 | 3% | 223 | 7% |
| Hospital Episode Statistics & Death Certificate & Self Report & Primary Care Data | 2 | 0% | 0 | 0% | 2 | 0% |
| Death Certificate & Self Report & Primary Care Data | 0 | 0% | 0 | 0% | 0 | 0% |
|  |  |  |  |  |  |  |
| Total events (Percentage of total VTEs) | 7169 | 100% | 4755 | 66% | 3155 | 44% |

Web Table 6 Count of hospital and self-report cases in UK Biobank broken down by source(s) and timing of report

| All data  (n =502520) | All Venous Thromboembolism | % | Deep Vein Thrombosis only | % | Pulmonary Embolism only | % |
| --- | --- | --- | --- | --- | --- | --- |
| PRIOR TO REGISTRATION |  |  |  |  |  |  |
| Hospital Episode Statistics only | 480 | 4% | 336 | 3% | 367 | 8% |
| Self Report only | 10051 | 74% | 8614 | 81% | 2705 | 59% |
| Hospital Episode Statistics & Self Report | 3111 | 23% | 1741 | 16% | 1480 | 33% |
| Total events  (Percentage of total VTEs) | 13642 | 49% | 10691 | 39% | 4552 | 16% |
| POST REGISTRATION |  |  |  |  |  |  |
| Hospital Episode Statistics only | 12428 | 89% | 4505 | 79% | 8191 | 94% |
| Self Report only | 1141 | 8% | 1012 | 18% | 338 | 4% |
| Hospital Episode Statistics & Self Report | 379 | 3% | 179 | 3% | 185 | 2% |
| Total events  (Percentage of total VTEs) | 13948 | 51% | 5696 | 21% | 8714 | 32% |

Web Table 7 Count of cases in the Primary Care cohort broken down by source(s) of report, excluding information from death certificates

| Primary Care subset  (n= 177358) | All Venous Thromboembolism | % | Deep Vein Thrombosis only | % | Pulmonary Embolism only | % |
| --- | --- | --- | --- | --- | --- | --- |
| EVENTS PRIOR TO REGISTRATION |  |  |  |  |  |  |
| Hospital Episode Statistics only | 149 | 3% | 105 | 3% | 113 | 7% |
| Self Report Report only | 3171 | 65% | 2826 | 74% | 772 | 46% |
| Hospital Episode Statistics & Self Report | 801 | 16% | 511 | 13% | 340 | 20% |
| Primary Care Data | 101 | 2% | 66 | 2% | 56 | 3% |
| Hospital Episode Statistics & Primary Care Data | 21 | 0% | 8 | 0% | 24 | 1% |
| Self Report & Primary Care Data | 320 | 7% | 178 | 5% | 179 | 11% |
| Hospital Episode Statistics & Self Report & Primary Care Data | 336 | 7% | 118 | 3% | 202 | 12% |
| Total events  (Percentage of total VTEs) | 4899 | 50% | 3812 | 39% | 1686 | 17% |
| EVENTS POST REGISTRATION |  |  |  |  |  |  |
| Hospital Episode Statistics only | 3652 | 76% | 1178 | 61% | 2487 | 82% |
| Self Report Report only | 355 | 7% | 318 | 16% | 109 | 4% |
| Hospital Episode Statistics & Self Report | 115 | 2% | 48 | 2% | 63 | 2% |
| Primary Care Data | 350 | 7% | 290 | 15% | 123 | 4% |
| Hospital Episode Statistics & Primary Care Data | 351 | 7% | 98 | 5% | 237 | 8% |
| Self Report & Primary Care Data | 8 | 0% | 8 | 0% | 1 | 0% |
| Hospital Episode Statistics & Self Report & Primary Care Data | 6 | 0% | 0 | 0% | 5 | 0% |
| Total events  (Percentage of total VTEs) | 4837 | 50% | 1940 | 20% | 3025 | 31% |

Web Table 8 : Count of incident cases in the Primary Care cohort broken down by source(s) of report including information from death certificates. (Cases prior to registration are not considered here, as there can be no prevalent events reported by death certificate by definition)

| Primary Care subset  (n= 177358) | All Venous Thromboembolism | % | Deep Vein Thrombosis only | % | Pulmonary Embolism only | % |
| --- | --- | --- | --- | --- | --- | --- |
| Hospital Episode Statistics only | 3397 | 66% | 1152 | 56% | 2269 | 66% |
| Death Certificate Only | 275 | 5% | 105 | 5% | 278 | 8% |
| Self Report only | 355 | 7% | 318 | 16% | 109 | 3% |
| Hospital Episode Statistics & Death Certificate | 255 | 5% | 26 | 1% | 218 | 6% |
| Hospital Episode Statistics & Self Report | 115 | 2% | 48 | 2% | 63 | 2% |
| Death Certificate & Self Report | 0 | 0% | 0 | 0% | 0 | 0% |
| Hospital Episode Statistics & Death Certificate & Self Report | 0 | 0% | 0 | 0% | 115 | 3% |
| Primary Care Data | 339 | 7% | 287 | 14% | 159 | 5% |
| Hospital Episode Statistics & Primary Care Data | 339 | 7% | 98 | 5% | 228 | 7% |
| Death Certificate & Primary Care Data | 11 | 0% | 3 | 0% | 8 | 0% |
| Self Report & Primary Care Data | 8 | 0% | 8 | 0% | 1 | 0% |
| Hospital Episode Statistics & Death Certificate & Primary Care Data | 12 | 0% | 0 | 0% | 9 | 0% |
| Hospital Episode Statistics & Self Report & Primary Care Data | 6 | 0% | 0 | 0% | 5 | 0% |
| Hospital Episode Statistics & Death Certificate & Self Report & Primary Care Data | 0 | 0% | 0 | 0% | 0 | 0% |
| Death Certificate & Self Report & Primary Care Data | 0 | 0% | 0 | 0% | 0 | 0% |
| Total events (Percentage of total incident VTEs) | 5112 | 100% | 2045 | 40% | 3462 | 68% |

Web Table 9 Kappa Concordance statistics from different data sources for VTE and subdefinition. Cohen’s kappa coefficient is given for each pair of sources, with overall Fliess Kappa used to compare all data sources

| VTE in primary data | | | | |
| --- | --- | --- | --- | --- |
|  | HES | ONS | GP | Self-report |
| HES |  | 0.06 | 0.29 | 0.33 |
| ONS |  |  | 0.02 | 0.01 |
| GP |  |  |  | 0.21 |
| Fliess Kappa | 0.192 |  | | |
| PE in primary data | | | | |
|  | HES | ONS | GP | Self-report |
| HES |  | 0.08 | 0.33 | 0.35 |
| ONS |  |  | 0.03 | 0.01 |
| GP |  |  |  | 0.33 |
| Fliess Kappa | 0.23 |  | | |
| DVT in primary data | | | | |
|  | HES | ONS | GP | Self-report |
| HES |  | 0.02 | 0.2 | 0.27 |
| ONS |  |  | 0.01 | 0 |
| GP |  |  |  | 0.14 |
| Fliess Kappa | 0.138 |  | | |
| VTE in all data | | | |  |
|  | HES | ONS | Self-report |  |
| HES |  | 0.06 | 0.32 |  |
| ONS |  |  | 0.01 |  |
| Fliess Kappa | 0.166 |  | |  |

Web Table 10a Concordance of reports of medication use between self-reports and GP records for all anti-coagulants and warfarin. Cohen’s kappa coefficient is given for each pair of sources
Table 10b Percentages of participants on anticoagulant medication and warfarin by source of case definition

| a) |  | |
| --- | --- | --- |
| All Anti-coagulants | Self-reported | No self report |
| GP-reported | 2727 | 33235 |
| No GP-report | 487 | 140909 |
| kappa | 0.11 | |
|  | | |
| Warfarin only |  |  |
|  | Self-reported | No self report |
| GP-reported | 1720 | 4878 |
| No GP-report | 286 | 170474 |
| kappa | 0.39 | |

|  |  |  |  |  |
| --- | --- | --- | --- | --- |
| Source of case definition | Self-reported medication use  (% cases) | | GP reported medication use  (% cases) | |
|  | Anti-coagulants | Warfarin | Anti-coagulants | Warfarin |
| Any | 10.8 | 9.1 | 50.6 | 32.4 |
| GP only | 13.9 | 12.5 | 37.9 | 26.8 |
| Self report only | 14.8 | 12.8 | 51.2 | 33.2 |
| HES only | 15.1 | 12.9 | 64.7 | 50.4 |
| ONS only | 4.7 | 3.1 | 36.3 | 12.7 |
| None | 1.4 | 0.7 | 18.9 | 2.5 |

Web Table 11 Demographic comparison between VTE case populations defined via the different data sources. Variables were based on the following UK Biobank fields: Manual labour (816), Shift working (826), Townsend deprivation index (189), House ownership (680), Number of cars in household (728). The Index of Mean Deprivation scores were only available for English participants from UK Biobank fields 26410-26417

|  | All UK Biobank  (n =502520) | | | | | Primary Care Subset  (n= 177358) | |
| --- | --- | --- | --- | --- | --- | --- | --- |
|  | All | Combined Data Cases | HES Data only cases | Self report only cases | Death Data only Cases | All | Primary Care  only cases |
| Gender (% Female) | 54.4 | 54.5 | 45.7 | 58.3 | 45.1 | 54.5 | 54 |
| Median Age (years) | 58.3 | 61.7 | 61.9 | 61.4 | 63.3 | 58.4 | 61.6 |
| White (%) | 94.6 | 96.1 | 96.5 | 96.0 | 96.6 | 95.7 | 97.3 |
| Not working (retired or unemployed) (%) | 43.1 | 60 | 60.4 | 59.9 | 68.8 | 44.1 | 61.1 |
| Higher Education (%) | 60.2 | 52.6 | 52.7 | 53 | 46.9 | 59.8 | 52.4 |
| Mean BMI | 27.4 | 29.2 | 29.5 | 29.2 | 30 | 27.5 | 29.2 |
| Current smokers (%) | 10.6 | 12.6 | 12.2 | 12.4 | 16.2 | 10.3 | 12.3 |
| Heavy manual work either "Usually" or "Always" (%) | 7.7 | 6.3 | 6.5 | 5.9 | 6.3 | 7.9 | 6.4 |
| shift work either "Usually" or "Always" (%) | 5.6 | 4.6 | 4.6 | 4.5 | 4 | 5.6 | 4.4 |
| Townsend deprivation index (mean) | -1.29 | -0.92 | -0.89 | -0.93 | -0.56 | -1.4 | -1.07 |
| Own house outright (%) | 51.5 | 55.8 | 56.8 | 55 | 54.4 | 52.9 | 57.5 |
| More than 1 car in household (%) | 48.8 | 41.7 | 40.7 | 42.4 | 32.5 | 48.3 | 41.2 |
| Wales Recruitment centre (%) | 4.1 | 4.6 | 3.9 | 4.9 | 4 | 10.3 | 14.1 |
| Scotland Recruitment centre (%) | 7.1 | 6.3 | 7 | 5.8 | 9.8 | 12.6 | 11.7 |
| London Recruitment centre (%) | 13.7 | 11.6 | 11.4 | 11.7 | 8.9 | 6.8 | 5.3 |
| *England participants only* |  |  |  |  |  |  |  |
| IMD (mean) | 17.68 | 19.69 | 19.77 | 19.72 | 21.32 | 17.68 | 19.36 |
| income score | 0.12 | 0.13 | 0.13 | 0.13 | 0.14 | 0.11 | 0.12 |
| employment score | 0.09 | 0.1 | 0.1 | 0.1 | 0.11 | 0.09 | 0.1 |
| health score | -0.09 | 0.03 | 0.03 | 0.03 | 0.13 | -0.01 | 0.08 |
| education score | 15.57 | 18.27 | 18.3 | 18.3 | 19.58 | 17.01 | 19.62 |
| housing score | 19.78 | 19.35 | 19.11 | 19.41 | 18.43 | 19.23 | 18.77 |
| crime score | -0.04 | 0.01 | 0.01 | 0.01 | 0.05 | -0.03 | 0.02 |
| living environment score | 18.74 | 19.16 | 19.23 | 19.09 | 20.41 | 18.49 | 19.03 |
